# Resting-state Networks in Tinnitus: A Scoping Review

**DOI:** 10.1007/s00062-022-01170-1

**Published:** 2022-05-12

**Authors:** Tori Elyssa Kok, Deepti Domingo, Joshua Hassan, Alysha Vuong, Brenton Hordacre, Chris Clark, Panagiotis Katrakazas, Giriraj Singh Shekhawat

**Affiliations:** 1 Ear Institute, University College London, London, UK; 2 College of Medicine and Public Health, Flinders University, Adelaide, Australia; 3 Innovation, IMPlementation and Clinical Translation (IIMPACT) in Health, Allied Health and Human Performance, University of South Australia, Adelaide, Australia; 4 Great Ormond Street Institute of Child Health, Department of Developmental Imaging and Biophysics, University College London, London, UK; 5 Zelus P.C., Athens, Greece; 6 Tinnitus Research Initiative, Regensburg, Germany

**Keywords:** Subjective tinnitus, Functional magnetic resonance imaging, Brain imaging, Neural networks, Auditory network, Review

## Abstract

Chronic subjective tinnitus is the constant perception of a sound that has no physical source. Brain imaging studies show alterations in tinnitus patients’ resting-state networks (RSNs). This scoping review aims to provide an overview of resting-state fMRI studies in tinnitus, and to evaluate the evidence for changes in different RSNs. A total of 29 studies were included, 26 of which found alterations in networks such as the auditory network, default mode network, attention networks, and visual network; however, there is a lack of reproducibility in the field which can be attributed to the use of different regions of interest and analytical methods per study, and tinnitus heterogeneity. Future studies should focus on replication by using the same regions of interest in their analysis of resting-state data, and by controlling adequately for potential confounds. These efforts could potentially lead to the identification of a biomarker for tinnitus in the future.

**Supplementary Information:**

The online version of this article (10.1007/s00062-022-01170-1) contains supplementary material, which is available to authorized users.

## Introduction

Tinnitus is the phantom perception of sound without an external source, and is commonly described as a ringing, hissing, whining, pure tone, or “cricket noise” [39, 66]. Tinnitus affects 10–15% of the adult population [39] and can have a profoundly negative effect on sleep, attention and overall quality of life [5]. Tinnitus can be classified as subjective or objective, pulsatile or non-pulsatile, and chronic (> 6 months) or recent onset (< 6 months) [5, 39, 67]. In subjective tinnitus, the noise can only be heard by the affected person, whereas in objective tinnitus the noise can be measured with specialized sound equipment [27]. Pulsatile tinnitus is almost always objective and has a specific, identifiable cause [41]. This review only considers chronic, subjective tinnitus as this type is hypothesized to be of central origin, but its exact mechanisms are unclear.

Despite increasing research into the field of tinnitus, the exact pathophysiology of tinnitus remains to be elucidated [5]. Observations that the tinnitus pitch is correlated to the frequency of maximum hearing loss in tinnitus patients [62] have led to the idea that deafferentation (e.g. due to hearing loss) could lead to neuroplasticity in an attempt to return neural activity to its usual homeostatic state [46, 60]. Neurons could become more susceptible to firing in response to spontaneous activity in the case of homeostatic strengthening of excitatory synapses or weakening of inhibitory synapses, referred to as increased gain. This firing could be interpreted as a sound, i.e. tinnitus [46]; however, not everyone with tinnitus has a visible hearing loss on normal audiograms, although a hidden hearing loss in the form of reduced neural output from the cochlea may be present in those cases [61].

Animal research has supported the plastic reorganization theory of tinnitus. Yang et al. [78] stated that plastic reorganization in the central auditory system and down-regulation of inhibitory synapses were observed in high frequency specific neurons in rat models that showed behavioral characteristics of high frequency tinnitus; however, tinnitus does not consistently arise under conditions that would be expected to create a tinnitus signal and not everyone with a hearing loss also develops tinnitus.

In a review on gain mechanisms and tinnitus, Sedley [65] concluded that increased gain can be induced by peripheral auditory insults but might not be sufficient to cause tinnitus. Rauschecker et al. [57] also postulated in their frontostriatal gating model of tinnitus that auditory lesion is not sufficient for tinnitus to arise. According to their model, tinnitus only occurs if the noise cancellation system, consisting of limbic-auditory connections mediated by the thalamus, breaks down. Tinnitus is also often accompanied by anxiety and depression [2] and attention networks play a role in tinnitus awareness [69], suggesting that the underlying mechanisms of tinnitus likely consist of multiple neural networks.

One method used to study neural networks is functional magnetic resonance imaging (fMRI). In fMRI, neural activity is not measured directly, but through the blood oxygen level-dependent (BOLD) signal [4, 56]. It is based on the observation that local blood flow increases with neural activity [59]. The unique fluctuations in BOLD responses are frequently used to compare patient groups with neurological conditions to neurotypical controls. BOLD fMRI has previously been utilized to demonstrate differences in functional networks in people with various neurological and psychiatric disorders (e.g. dementia [58], Alzheimer’s disease [35, 47, 68], depression [34] and schizophrenia [72]).

A popular method for the study of functional networks is resting-state fMRI (rs-fMRI). Rs-fMRI quantifies the temporal dependence of neural activity patterns between anatomically separated regions when the subject is not engaged in any task. BOLD signal fluctuations that are correlated in anatomically separated regions can be used to infer functional connectivity between those regions [72]. Early work in this field revealed a high correlation between spontaneous neural activation patterns in the motor network [8, 9] with later studies replicating this for other networks such as the primary visual network and the auditory network [72]. Rs-fMRI investigates low frequency oscillations (~ 0.01–0.1 Hz) of the fMRI time series, which may be confounded by cardiac and respiratory oscillations, although it is generally accepted that these resting-state fMRI patterns have a neuronal basis as they reflect the traditional known systems such as the motor and visual systems [8, 9, 72].

Rs-fMRI research has given rise to the identification of several prominent resting-state networks (RSN) which are consistently identified, regardless of different subject groups, methods of analysis and scanning protocols. These are the sensorimotor network, visual and extrastriate visual network, insular-temporal/anterior cingulate cortex (ACC) (saliency) network, left and right lateralized frontoparietal networks (attention), default mode network (DMN), and a frontal executive function network [72].

The RSN that has received the most attention in research into cognitive dysfunctioning is the DMN, which is characterized by being more active at rest than during task state. It comprises posterior cingulate cortex (PCC), precuneus, medial prefrontal cortex (mPFC), and inferior parietal cortical regions [6, 25, 71]. As tinnitus is mainly experienced during rest, it has been hypothesized that alterations in the DMN could be associated with tinnitus [15, 42].

Other RSNs have been identified than the eight presented above. Most notably of relevance here is the auditory RSN. Cordes et al. [24] asked subjects to perform an auditory text listening task and compared the activity map derived from the task-based fMRI scan to an activity map created using rs-fMRI using the auditory cortex as a region of interest. They found the distribution of the auditory task activity was very similar to the resting-state functional connectivity network in auditory regions. Evidence taken together suggests characteristics of functional connectivity maps reflect how networks appear during the relevant active tasks [24].

Other networks that are often mentioned are the dorsal attention network (DAN) and ventral attention network (VAN), which can be confused with the lateralized frontoparietal (FP) networks. Data-driven clustering of networks shows an overlap in some individuals between the FP networks and DAN, but generally they can be distinguished by the FP covering prefrontal cortex and intraparietal sulcus and the DAN covering a dorsal premotor strip and frontal eye fields [33].

Husain and Schmidt [42] reviewed six studies on tinnitus and rs-fMRI focusing on the DMN, visual RSN, auditory RSN, the DAN and the limbic network. They found that the DMN-limbic and the auditory-limbic functional connectivity was increased in tinnitus and may be correlated with tinnitus-related distress. New evidence on RSNs in tinnitus has been gathered since the review of Husain and Schmidt [42] and therefore a comprehensive review is needed to bring together current evidence from rs-fMRI in tinnitus patients. This scoping review aims to present an overview of the research in this area and to answer the question: What has rs-fMRI revealed about resting-state networks in tinnitus patients?

## Methods

### Search Strategy

A scoping review was undertaken using the framework proposed by Arksey and O’Malley [3]. In a scoping review, the goal is to map all the available evidence in a research area and to highlight gaps in the existing literature, without excluding studies based on their quality [3, 51]. The online databases PubMed, Embase, and Web of Science Core Collection (WoS CC) were searched from inception until 22/01/2021 using the following search term:

(“fmri”[tiab] OR “mri”[tiab] OR “magnetic resonance imaging” [tiab] OR “functional connectivity”[tiab] OR “resting state”[tiab]) AND “tinnitus”[ti]

### Inclusion Criteria

To be included, studies had to be primary research studies, in which subjects with chronic (> 6 months), subjective tinnitus underwent resting-state fMRI scanning. All subjects had to be adults (> 18 years old). Only studies written in English were included. Studies were excluded if they were case studies, review studies, brain imaging studies that did not use rs-fMRI, treatment studies, studies in which subjects had pulsatile tinnitus, animal studies, studies with children, and hospital diagnostic studies. Studies that used rs-fMRI combined with other types of MRI were included. There were no inclusion criteria regarding the presence of a control group.

All papers were screened for inclusion by two independent reviewers (T. Kok and D. Domingo) in both abstract stage and full-text stage. Any clashes between the two reviewers were resolved through a discussion between the two reviewers.

### Data Charting

The included papers were taken to the data charting stage. Information was extracted about the study design, participant characteristics, results, and MRI scanning protocols and logged in Excel spreadsheets. All data were extracted as mean ± standard deviation (M ± SD) unless otherwise specified.

### Data Analysis

Papers were grouped by their method of analyzing the resting-state data and summarized in tables. Then, papers were also grouped by which networks were implicated in their findings for the discussion. A brain template was created with the most important regions for each relevant resting-state network depicted on it (Fig. 2). These regions were selected based on the RSN literature. To summarize the findings from seed-based functional connectivity studies, if at least one study found an increased or decreased connection between two regions included on the brain template, a solid or dotted line was drawn between the regions. Findings related to any other regions were not presented on the figure, but are available in the results tables, as the number of different regions implicated in total was too high to include all of them in one figure.

For auditory findings specifically, a second figure was created by tallying how often an increased or decreased connection was found with an auditory region-of-interest (Fig. 3).

## Results

### Search Results

**Fig. 1:**
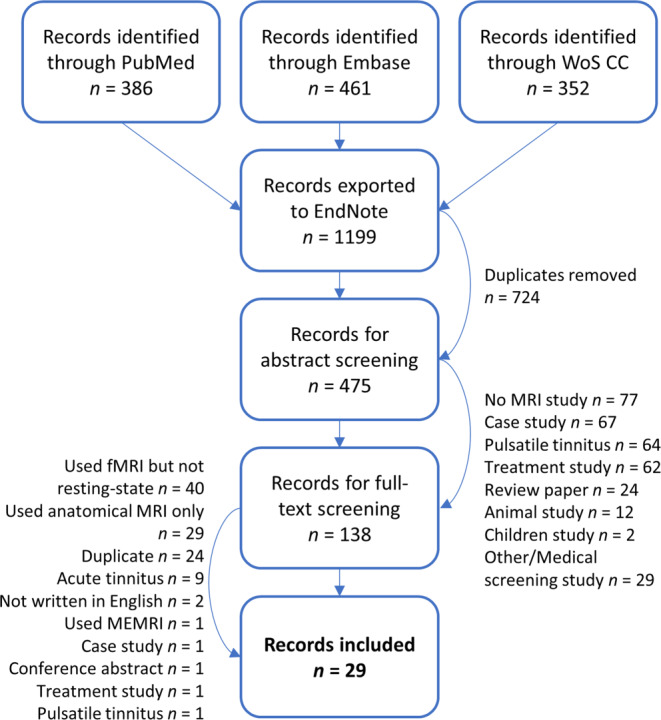
Flowchart of study selection. *WoS CC* World of Science Core Collection, *MEMRI* manganese-enhanced MRI

There were 386 hits in PubMed, 461 hits in Embase, and 352 hits in World of Science Core Collection. All records were exported to the EndNote X9 (Clarivate Analytics, Philadelphia, PA, USA) reference management software. Fig. 1 shows the flowchart for study selection.

### Design of Studies

The 29 studies had total sample sizes ranging from 12–105 participants (M 50.69 ± SD 22.37), with sample sizes for tinnitus groups ranging from 6–50 participants (M 23.47 ± SD 10.25), and control groups ranging from 6–55 participants (M 23.48 ± SD 11.81). From 29 studies, 25 studies defined chronic subjective tinnitus as at least 6 months (confirmed either within the manuscript or through confirmation via email with the authors), 3 studies defined chronic as at least 1 year, and 1 study defined chronic tinnitus as greater than 2 years.

Different methods to analyze the rs-fMRI data were found in the included studies. The most common method for building a functional connectivity map was seed-based correlation analysis (SCA). In SCA, the linear correlation is calculated between the time series of a seed region/voxel (an a priori specified “region of interest”), and every other voxel in the brain. Eighteen out of 29 studies or 62% used SCA to analyze functional connectivity patterns and 3 studies used Granger causality analysis, which is also a seed-based method but deploys a statistical method from the field of economics to assess directional aspects of connectivity [18, 32].

Three studies used regional homogeneity (ReHo) analysis, which can be used to quantify local synchronization in neighboring voxels and is a measure of local neural activity coherence during resting-state [79]. Increased ReHo values reflect increased local synchrony, which could be interpreted as increased coherence of spontaneous neural activity [79].

Four studies used amplitude of low-frequency fluctuation calculations (ALFF) to examine resting-state fMRI data. ALFF is an algorithm used to measure the intensity of intrinsic brain activity at a regional level. Resting-state ALFF could reflect abnormal changes in activity in various neurological disorders [17]. ALFF examines brain activity in specific frequency bands rather than across a broad, low-frequency band between 0.01 and 0.1 Hz, as is predominantly used in resting-state fMRI [21].

One study used voxel-mirrored homotopic connectivity (VMHC), which measures synchrony between a voxel in one hemisphere and its mirrored counterpart in the opposite hemisphere, and it can be used to study interhemispheric functional connectivity [20].

Three studies used independent component analysis (ICA), which is a data-driven method in which it is not necessary to define a region of interest in advance [52]. ICA is a mathematical technique to separate a set of data into components based on statistical independence. This data-driven technique can be applied to rs-fMRI data to identify spatially distinct resting-state networks [47]. The user has to determine the number of independent components to create from the data, which is known to have an effect on the results [45].

A final proof-of-concept study used cyclicity analysis, which studies leader-follower relationships between two signals in a time series.

### Study Findings

#### Seed-based Correlation Analysis: Non-directional

Fifteen studies used non-directional SCA to analyze the resting-state patterns in tinnitus patients. Regions of interest (ROI) were predefined in these studies, and the correlations between the rs-fMRI time series of these ROIs and all other voxels in the brain were calculated. The resulting functional connectivity map of the chosen seed regions was then compared between tinnitus and control groups.

A wide range of ROIs were investigated, consisting of auditory and non-auditory seed regions. Non-auditory seed regions were often located in a known resting-state network, such as the DMN, DAN, VAN, visual network, sensorimotor network, or cognitive/control network. ROIs were selected in different ways, most often using WFU_PickAtlas software (NITRC – NeuroImaging Tools & Resources Collaboratory, https://www.nitrc.org/projects/wfu_pickatlas/).

**Fig. 2:**
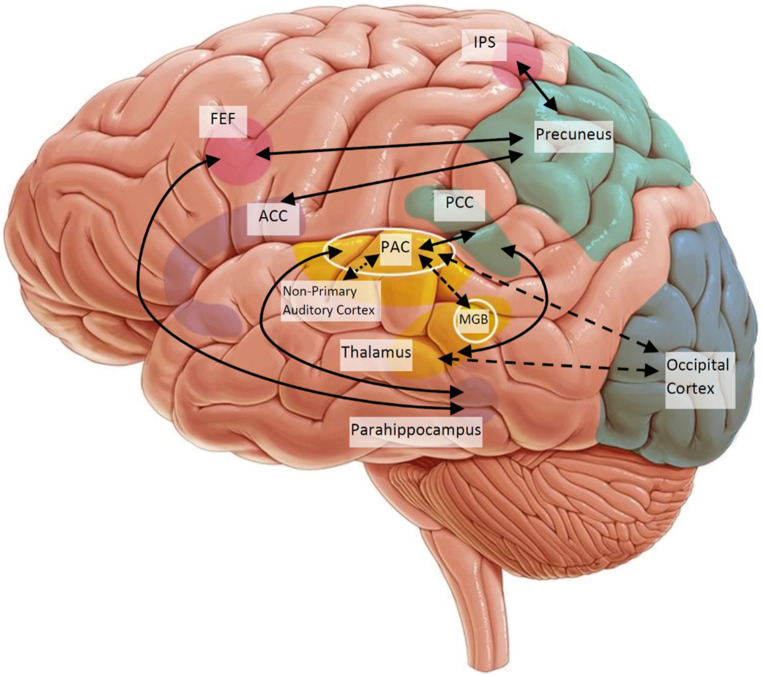
Schematic of findings of seed-based studies. Solid lines represent increased functional connectivity between regions of the brain, whereas dotted lines represent decreased functional connectivity in tinnitus groups compared to control groups. A line was drawn between two regions if at least one paper found this increased/decreased connection. Only the results relevant to the regions on the template are presented in this overview. *Yellow* auditory network, *aqua* default mode network, *pink* dorsal attention network, *purple* limbic system, *blue* visual network. Please note some of the presented structures are internal to the brain and their location is therefore an approximation on this schematic *IPS* intraparietal sulcus, *FEF* frontal eye fields, *ACC* anterior cingulate cortex, *PCC* posterior cingulate cortex, *MGB* medial geniculate body. (Image template copyright: Kenhub GmbH, illustrator: Paul Kim, Ocran [55] (permission for use granted))

Fig. 2 shows an overview of the findings of SCA studies.

All but two studies [12, 48] showed altered functional connectivity networks in tinnitus patients. Table 1 shows an overview of the studies’ findings; Table S1 is the extended version with detailed information about each study’s design.

**Table 1:** Main findings of non-directional seed-based studies

Study	Sample size	Hearing loss	Control matching	ROI/seed	Findings of increased ↑ or decreased ↓ FC (tinnitus vs. control)	Networks implicated
Berlot et al. 2020 [7]	Tinnitus *n* =6 Control *n* =6	Yes, matched	Age Sex Hearing thresholds Handedness	PAC, Non-PAC, MGB, IC	↓ PAC <> non-PAC ↓ PAC <> MGB	Auditory
Chen et al. 2017 [14]	Tinnitus *n* = 40 (20 depressive) Control *n* = 23	Normal audiogram	Age Sex Hearing thresholds Years of education SAS/SDS scores	L Amygdala, R Amygdala	Tinnitus (depressed + non-depressed) vs. controls: ↑ L + R Amygdala <> L PoCG ↓ L + R Amygdala <> L STG ↓ L Amygdala <> L MFG ↓ L Amygdala <> R PCC ↓ R Amygdala <> R MFG ↓ R Amygdala <> R SFG See Table S1 for other group comparisons	Prefrontal-cingulate-temporal circuit DMN Attention Somatosensory Visual
Chen et al. 2018 [15]	Tinnitus *n* = 40 Control *n* = 41	Normal audiogram	Age Sex Hearing thresholds Years of education Handedness	ACC, PCC	↑ ACC <> L Precuneus* ↑ PCC <> R mPFC** * positively correlated with tinnitus duration (r = 0.451, *p* = 0.007) ** positively correlated with tinnitus distress (r = 0.411, *p* = 0.014)	DMN
Chen et al. 2018 [16]	Tinnitus *n* = 31 Control *n* = 40	Normal audiogram	Age Sex Years of education Hearing thresholds SDS and SAS scores Brain parenchyma volume Handedness	Rostral ACC, dorsal ACC	↑ rACC <> L Precuneus* ↑ rACC <> R PoCG ↑ rACC <> R Putamen ↑ dACC <> R STG ↑ dACC <> R IPL** ↑ dACC <> R OFC ↑ dACC <> R mPFC ↓ rACC <> L Calcarine cortex ↓ dACC <> R Fusiform gyrus * positively correlated with tinnitus severity (r = 0.507, *p* = 0.008). ** positively correlated with tinnitus severity (r = 0.447, *p* = 0.022)	Auditory DMN Visual Executive functions Somatosensory
Chen et al. 2018 [22]	Tinnitus *n* = 35 Control *n* = 50	Normal audiogram	Age Sex Years of education Hearing thresholds SDS & SAS scores Brain parenchyma volume Grey and white matter volume Handedness	PCC	↑ PCC <> R mPFC* * Correlated with the poorer Trail Making Test-B scores (r = 0.474, *P* = 0.008) but not with tinnitus diagnostics	DMN
Feng et al. 2018 [28]	Tinnitus *n* = 28 Control *n* = 29	Normal audiogram	Age Sex Years of education Hearing thresholds Brain parenchyma volume Grey and white matter volume Handedness	Cerebellum (9 seeds)	↑ L Crus I <> L PHG ↑ R Crus I <> R IOG ↑ R Crus II <> R IOG ↑ L Lobule VIIb <> R STG* ↑ R Lobule VIIb <> L PCG ↑ Vermis <> R STG** *positively correlated with THQ scores (r = 0.577, *p* = 0.004). **positively correlated with the THQ score (r = 0.432, *p* = 0.039)	Auditory Limbic system Visual
Henderson-Sabes et al. 2019 [37]	Tinnitus *n* = 15 Control *n* = 15	Unilateral deafness, matched	Age Sex Handedness Hearing thresholds Duration of deafness	L + R HG, L + R caudate nucleus	↑ L caudate nucleus <> L HG ↑ L caudate nucleus <> R SMA	Auditory Limbic system Motor DMN Visual DAN
Hinkley et al. 2015 [40]	Tinnitus *n* = 15 Control *n* = 15	Yes, not matched, hearing loss included as covariate in analysis	Age Sex	L + R PAC, L + R dorsal striatum, L + R caudate head, L + R NAc	↑ L PAC <> R STG ↑ L PAC <> L MTG ↑ L PAC <> SFG ↑ L PAC <> posterior cerebellum ↑ L PAC <> PHG ↑ L PAC <> L lingual gyrus ↑ R PAC <> L MTG ↑ R PAC <> L SFG ↑ R PAC <> L MOG ↑ R PAC <> R PoCG ↑ Striatal ROIs <> frontal, temporal & occipital regions (See Table S1) ↓ L + R dorsal striatum <> L + R lingual gyrus ↓ L dorsal striatum <> L culmen ↓ R caudate head <> L culmen ↓ R caudate head <> R lingual gyrus ↓ L NAc <> R STG ↓ L NAc <> R culmen ↓ L NAc <> L lingual gyrus ↓ L NAc <> L IPL	Auditory Limbic system Visual DMN DAN
Job et al. 2020 [44]	Tinnitus *n* = 19 Control *n* = 19	Yes, not matched, hearing loss included as covariate in analysis	Age Sex	L + R HG, L + R MGB, L + R IC, *n* = 5 operculum ROIs, *n* = 7 whole brain rs networks	↑L HG <> PCC ↑ L &R IC <> R SPL ↑ R operculum <> R SFG ↑ Posterior R operculum <> L SFG ↑ Posterior R operculum <> L IPL Whole brain RSN analysis found enhanced FC with sensorimotor-auditory network & frontoparietal network	Auditory DMN Sensorimotorauditory Frontoparietal
Zhang et al. 2015 [80]	Tinnitus *n* = 31 Control *n* = 33	Normal audiogram	Age Sex Hearing thresholds Years of education Handedness	L + R thalamus	↑ L thalamus <> R angular gyrus ↑ L thalamus <> R MCC ↑ L thalamus <> L CPL ↑ R thalamus <> L PCC ↑ R thalamus <> L + R CPL ↓ L thalamus <> R MTG* ↓ L thalamus <> R MOC ↓ L thalamus <> L MFG ↓ L thalamus <> R PCG ↓ L thalamus <> L + R calcarine cortex ↓ R thalamus <> L STG** ↓ R thalamus <> L amygdala ↓ R thalamus <> R SFG ↓ R thalamus <> L PCG ↓ R thalamus <> L MOG *Negatively correlated with THQ score (r = −0.482, *p* = 0.011). **Negatively correlated with tinnitus duration (r = −0.454, *p* = 0.017)	Auditory Visual DMN
Schmidt et al. 2017 [64]	MRTIN *n* = 13 MLTIN_1 *n* = 12 MLTIN_2 *n* = 17 BLTIN *n* = 15 Controls NH *n* = 15 Controls HL *n* = 13	Yes, matched	Age Sex Hearing thresholds (in the case of the HL control group)	L + R PAC, DMN (combined: mPFC, PCC), DAN (combined: L + R IPS, L + R FEF)	For all tinnitus groups vs. controls or long- vs. short-term tinnitus: ↑ DAN <> precuneus ↑ DAN <> region near L PCG (unspecified); ↓ DMN <> precuneus ↓ DMN <> FMC ↓ DMN <> & lateral SOC No differences were found between mild and bothersome tinnitus subgroups	DMN DAN
Wineland et al. 2012 [76]	Tinnitus *n* = 18 Controls *n* = 23	Yes, not matched	Sex	58 spherical seed-regions to reflect 7 networks: DAN, VAN, DMN, auditory, cognitive, visual, somatosensory	None found	None found
Burton et al. 2012 [12]	Tinnitus *n* = 17 Controls *n* = 17	Yes, not matched	Age	Auditory (L + R PAC), Visual (R V1, L cuneus), Somatosensory (R PoCG, L PO), DAN (L + R IPS, L FEF, R VIS), VAN (R TPJ, R STS), Attention control (R MFG, R AI, L + R IFG)	↑ L IFG <> R AI ↓ L + R PAC <> occipital pole ↓ L + R PAC <> L POS ↓ L + R PAC <> calcarine sulcus ↓ L + R PAC <> cuneus ↓ L + R PAC <> lingual gyri ↓ R V1 <> L STF ↓ R V1 <> L sulcal AC ↓ R V1 <> L rostral insula ↓ R V1 <> L IFG ↓ R AI <> L + R mOC ↓ R AI <> L + R lOC ↓ L IFG <> mOC	Auditory Visual Attention control
Minami et al. 2018 [53]	Tinnitus HL *n* = 18 Tinnitus NH *n* = 11 Control *n* = 19	Yes, not matched	Not given	HG, planum temporale, planum polare, operculum, insular cortex, STG	ROI names and statistics are not legible in figures due to poor image quality. According to authors FC in auditory ROIs was weakened in tinnitus	Auditory
Lee et al. 2012 [48]	Tinnitus *n* = 16 Control *n* =0	Yes	Intra-subject comparison	58 spherical seed regions in 7 networks: DMN, DAN, VAN, cognitive/control, auditory, visual, somatosensory	Participants modulated their tinnitus using orofacial maneuvers and served as their own baseline, but no differences were found	None found

*<>* functional connectivity, *ROI* region of interest, *PAC* primary auditory cortex, *MGB* medial geniculate body, *IC* inferior colliculus, *FC* functional connectivity, *L* left, *R* right, *DMN* default mode network, *ACC* anterior cingulate cortex, *PCC* posterior cingulate cortex, *mPFC* medial prefrontal cortex, *IPL* inferior parietal lobule, *STG* superior temporal gyrus, *HG* Heschl’s gyrus, *SMA* supplementary motor area, *DAN* dorsal attention network, *NA* nucleus accumbens, *THI* Tinnitus Handicap Inventory, *rs* resting-state, *SFG* superior frontal gyrus, *MCC* middle cingulate cortex, *CPL* cerebellar posterior lobe, *MOC* middle orbitofrontal cortex, *PCG* precentral gyrus, *MRTIN* mild recent tinnitus, *MLTIN* mild long-term tinnitus, *BLTIN* bothersome long-term tinnitus, *NH* no hearing loss, *HL* hearing loss, *IPS* intraparietal sulcus, *FEF* frontal eye field, *FMC* frontal medial cortex, *SOC* superior occipital cortex, *VAN* ventral attention network, *V1* primary visual cortex, *PO* parietal operculum, *VIS* ventral intraparietal sulcus, *TPJ* temporoparietal junction, *STS* superior temporal sulcus, *AI* anterior insula, *POS* parietal occipital sulcus, *mOC* medial occipital cortex, *lOC* lateral occipital cortex

#### Seed-based Correlation Analysis: Directional (Granger Causality Analysis)

Three studies used Granger Causality Analysis (GCA) to investigate directional connectivity in tinnitus patients. One study [18] selected ROIs based on degree centrality (a graph theory based, data-driven method) whereas the other two studies [19, 77] selected ROIs manually. Table 2 is an overview of GCA findings; extended information is available in Table S1.

**Table 2:** Main findings of directional seed-based studies

Study	Sample size	Hearing loss	Control Matching	ROI/seed	Findings of increased ↑ or decreased ↓ FC (tinnitus vs. control)	Networks implicated
Chen et al. 2016 [18]	Tinnitus *n* = 24 Control *n* = 22	Normal audiogram	Age Sex Hearing thresholds Years of education	L + R SFG	↑L SFG → L OFC* ↑L SFG → L PCG ↑L SFG → L PLC ↑L SFG → R MOG ↑R SFG → R SMA** * Positively correlated with THQ scores (r = 0.504, *p* = 0.020). ** Positively correlated with THQ scores (r = 0.526, *p* = 0.014)	Motor Visual Frontal Somatosensory
Chen et al. 2017 [19]	Tinnitus *n* = 26 Control *n* = 23	Normal audiogram	Age Sex Hearing thresholds Handedness Years of education	L + R amygdala, L + R hippocampus	↑ L amygdala → L STG* ↑ L + R amygdala → L ACC ↑ L amygdala → R angular gyrus ↑ L amygdala → L precuneus ↑ L amygdala ← R MFG ↑ L + R amygdala ← L MTG ↑ L amygdala ← L IFG ↑ L amygdala ← L PoCG ↑ R amygdala → R MFG ↑ R amygdala → R STG** ↑ R amygdala → R SMG ↑ R amygdala ← L MTG ↑ R amygdala ← R PoCG ↑ L Hippocampus → L MTG ↑ L Hippocampus → L PoCG ↑ L Hippocampus ← R SFG ↑ L Hippocampus ← L Parahippocampal gyrus ↑ L Hippocampus ← L Insula ↑ R hippocampus → L TTG*** ↑ R hippocampus → R MTG ↑ R hippocampus → R PoCG ↑ R hippocampus ← L MTG ↑ R hippocampus ← L + R MFG ↑ R hippocampus ← L angular gyrus ↓ L amygdala → L PLC ↓ R amygdala → R PLC ↓ L hippocampus → L MOG ↓ R hippocampus → R MOG * positively correlated with THQ scores (r = 0.570, *p* = 0.005). ** positively correlated with THQ scores (r = 0.487, *p* = 0.018). *** positively correlated with tinnitus duration (r = 0.452, *p* = 0.030)	Auditory Limbic system DMN DAN Executive control of attention
Xu et al. 2019 [77]	Tinnitus *n* = 50 Control *n* = 55	Normal audiogram	Age Sex Hearing thresholds Years of education	L + R NAc	↑L NAc → L IFG ↑ L NAc ← R MFG* ↑ L Nac ← R MTG. ↑ R NAc → L MFG** ↑ R NAc → R OFC*** ↑ R NAc ← R IFG ↑ R NAc ← R MTG ↓ L NAc → L Cuneus ↓ R NAc → R Cuneus * Positively correlated with THQ scores (r = 0.626, *p* < 0.001). **Positively correlated with THQ scores (r = 0.357, *p* = 0.015). *** Positively correlated with tinnitus duration (r = 0.599, *p* < 0.001)	Frontostriatal circuit Limbic system

<> functional connectivity, *ROI* region of interest, *L* left, *R* right, *SFG* superior frontal gyrus, *OFC* orbitofrontal cortex, *PCG* precentral gyrus, *PLC* posterior lobe of cerebellum, *MOG* middle occipital gyrus, *SMA* supplementary motor area, *THQ* Tinnitus Handicap Questionnaire, *DMN* default mode network, *DAN* dorsal attention network, *STG* superior temporal gyrus, *TTS* transverse temporal gyrus, *NAc* nucleus accumbens, *IFG* inferior frontal gyrus, *MFG* middle frontal gyrus, *MTG* Middle temporal gyrus

#### Non-seed-based Studies

Eight studies used alternative methods of analysis, including amplitude of low-frequency fluctuations (ALFF), regional homogeneity (ReHo), voxel-mirrored homotopic connectivity (VMHC), and cyclicity analysis. Table 3 below shows the main findings of these studies; Table S1 shows an extended overview.

**Table 3:** Main findings of non-seed-based studies

Study	Sample size	Hearing loss	Control Matching	Analysis	Findings of increased ↑ or decreased ↓ values/FC (tinnitus vs. control)	Networks implicated
Cai et al. 2019 [13]	Tinnitus *n* = 16 Control *n* = 15	Normal audiogram	Age Sex Years of education	smALFF & seed-based FC	↑ smALFF values in L HAC, which was positively correlated with tinnitus duration (r = 0.778, *p* > 0.001), Tinnitus Handicap Inventory Score (r = 0.682, *p* = 0.004), and Self-Rating Depression Score (r = 0.694, *p* = 0.003); ↑L HAC <> a wide range of regions (see Table S1); ↑ smALFF value in R Inferior Colliculus, not correlated to any clinical characteristics	Auditory Motor DAN Executive control Emotion
Chen et al. 2014 [17]	Tinnitus *n* = 31 Control *n* = 32	Normal audiogram	Age Sex Hearing thresholds Handedness Years of education	ALFF	↑ ALFF values in R MTG, R SFG, and R angular gyrus. ↓ ALFF values in L cuneus, R MOG, and L + R thalamus	Auditory DMN Visual
Chen et al. 2015 [21]	Tinnitus *n* = 39 Control *n* = 41	Normal audiogram	Age Sex Hearing thresholds Years of education	ALFF/fALFF	↑ ALFF values in R SFG*, R MTG, R angular gyrus, L IFG, and R SMG ↓ ALFF values in L + R MOG ↑ fALFF values in L SFG** and R SMG ↓ fALFF values in L + R MOG. *Positively correlated with THQ score (r = 0.446, *p* = 0.007) and tinnitus duration (r = 0.544, *p* = 0.001). ** Positively correlated with THQ score (r = 0.466, *p* = 0.005) and tinnitus duration (r = 0.526, *p* = 0.001)	Auditory DMN Visual
Han et al. 2018 [36]	Tinnitus *n* = 25 Control *n* = 25	Normal audiogram	Age Sex Hearing thresholds Years of education HQ score	ReHo, fALFF & seed-based FC	↑ ReHo values in R MTG & R cuneus ↓ ReHo values in R MFG & cerebellar anterior lobe ↑ fALFF values in R MTG ↓ R MTG <> R MFG* ↓ R MTG <> R lingual gyrus ↓ R MTG <> R cerebellar posterior lobe ↓ R cuneus <> R MTG * Positively correlated with Tinnitus Handicap Inventory score (r = 0.675, *p* = 0.001)	Auditory DMN Visual
Chen et al. 2015 [23]	Tinnitus *n* = 29 Control *n* = 30	Normal audiogram	Age Sex Hearing thresholds Years of education	ReHo & seed-based FC	↑ ReHo values in L + R AI, L IFG and R SMG ↓ ReHo values in L cuneus ↑ L AI <> L MFG* ↑ L AI <> R ITG ↑ L AI <> R precuneus ↑ R AI <> R MFG** ↑ R AI <> R STG ↑ R AI <> L precuneus ↑ R AI <> L PCC ↑ L IFG <> R MFG ↑ L IFG <> R ITG ↑ L IFG <> R ACC ↑R SMG <> L IFG ↑R SMG <> R OFC. * Positively correlated with THQ score (r = 0.459, *p* = 0.012). ** Positively correlated with THQ score (r = 0.479, *p* = 0.009)	Attention DMN Visual
Gentil et al. (2019) [31]	Tinnitus *n* = 19 Control *n* = 16	Yes, mild, not matched	Age Handedness Years of education	ReHo & correlation analysis	↓ ReHo values in cluster between STG/MTG overlapping auditory cortex Significant correlations between tinnitus clinical characteristics and several brain regions (see Table S1 for specifics)	Auditory
Chen et al. 2015 [20]	Tinnitus *n* = 28 Control *n* = 30	Normal audiogram	Age Sex Hearing thresholds Years of education	VMHC & correlation analysis	↑ VMHC values in bilateral MTG, MFG & SOG Significant positive correlation between VMHC values in tinnitus patients and clinical characteristics: THQ score & TTG (auditory cortex) (r = 0.63775); THQ score & STP (r = 0.71195); THQ score & PCG (r = 0.64225); THQ score & CC (r = 0.65234); Uncus & tinnitus duration (r = 0.62026)	Auditory Visual Motor DMN Limbic system
Zimmerman et al. 2019 [81]	Tinnitus *n* = 32 Control *n* = 15	Mild-moderate high frequency hearing loss	Age BDI and BAI scores	Cyclicity analysis	Cyclicity analysis was able to differentiate between tinnitus and control groups with 58–67% accuracy. Temporal patterns in rs-fMRI data were less consistent in tinnitus patients than in controls. Twenty regions contributed the most towards distinguishing the tinnitus and controls groups using machine learning classification methods (see Table S1 for specifics)	Auditory DMN DAN VAN Attention control Limbic system

<> functional connectivity, *smALFF* smoothed mean amplitude of low-frequency fluctuations, *HAC* higher auditory cortex, *DAN* dorsal attention network, *FC* functional connectivity, *ALFF* amplitude of low-frequency fluctuations, *MTG* middle temporal gyrus, *SFG* superior frontal gyrus, *MOG* middle occipital gyrus, *DMN* default mode network, *fALFF* fractional amplitude of low-frequency fluctuations, *IFG* inferior frontal gyrus, *SMG* supramarginal gyrus, *ReHo* regional homogeneity, *MFG* middle frontal gyrus, *AI* anterior insular cortex, *ITG* inferior temporal gyrus, *STG* superior temporal gyrus, *PCC* posterior cingulate cortex, *ACC* anterior cingulate cortex, *VMHC* voxel-mirrored homotopic connectivity, *SOG* superior occipital gyrus, *TTG* transverse temporal gyrus, *STP* superior temporal pole, *PCG* precentral gyrus, *CC* calcarine cortex, *HQ* Hyperacusis Questionnaire, *BDI* Beck’s Depression Inventory, *BAI* Beck’s Anxiety Inventory, *THQ* Tinnitus Handicap Questionnaire

#### Data-driven Studies: Independent Component Analysis (ICA)

Three studies used ICA followed by seed-based FC analysis. ICA is a mathematical technique to separate a set of data into components based on statistical independence. This data-driven technique can be applied to rs-fMRI data to identify spatially distinct resting-state networks [47]. This means the user does not have to define an a priori region of interest as in seed-based studies. The studies below visually selected the auditory component based on the ICA outcome and used this as a region of interest in the seed-based FC analysis. The main findings are presented in Table 4 below; see Table S1 for an extended version.

**Table 4:** Main findings of studies using Independent Component Analysis

Study	Sample size	Hearing loss	Control Matching	Analysis	Main finding (tinnitus vs. control)	Network implicated
Davies et al. 2014 [26]	Tinnitus *n* = 12 Control *n* = 11	Mild-moderate high-frequency hearing loss	Age Sex Hearing thresholds BDI & BAI scores	ICA + seed-based FC Total *n* components created: 23	None found (result did not survive multiple comparison correction)	None found
Maudoux et al. 2012 [50]	Tinnitus *n* = 13 Control *n* = 15	Mild-to-severe hearing loss, not matched	Age Sex	ICA + seed-based FC Total *n* components created: 30 Auditory component: L + R Heschl’s Gyrus, L + R STG, and L + R Insula	↑ Auditory component <> L + R PHG ↑ Auditory component <> L + R brainstem/cerebellum ↑ Auditory component <> L PCG ↑ Auditory component <> L STG ↑ Auditory component <> L IFG ↑ Auditory component <> R basal ganglia ↑ Auditory component <> R PFC ↑ Auditory component <> L PoCG ↑ Auditory component <> R OFC ↑ Auditory component <> R IPL ↓ Auditory component <> L SFG ↓ Auditory component <> L Fusiform gyrus ↓ Auditory component <> R STG ↓ Auditory component <> L + R Occipital cortex ↓ Auditory component <> L PFC	Auditory Attention Emotion Memory Visual
Schmidt et al. 2013 [63]	Tinnitus *n* = 12 NH control *n* = 15 HL control *n* = 13	Moderate-severe high-frequency HL	Age Sex Hearing thresholds (for HL control group)	ICA + seed-based FC Total *n* components created: 30 Auditory component: L + R PAC	↑ Auditory component <> L Lingual Gyrus (TIN > NH) ↑ Auditory component <> L Parahippocampus (TIN > NH) ↑ DAN <> R Parahippocampus (TIN > HL) ↑ DMN <> R Fusiform Gyrus (TIN > HL) ↑ DMN <> R Lingual Gyrus (TIN > HL) ↓ DAN <> R SMG (HL > TIN) ↓ DMN <> L Precuneus (HL > TIN) ↓ DMN <> L PCG (HL > TIN) ↓ DMN <> L Cerebellum (HL > TIN) ↓ DMN <> L Cerebellar Vermis (HL > TIN) ↓ DMN <> and R Precuneus (NH > TIN)	Auditory DAN DMN Motor Limbic system

*BDI* Beck’s Depression Inventory, *BAI* Beck’s Anxiety Inventory, *ICA* independent component analysis, *FC* functional connectivity, *L* left, *R* right, *STG* superior temporal gyrus, *PHG* parahippocampal gyrus, *PCG* precentral gyrus, *IFG* inferior frontal gyrus, *PFC* prefrontal cortex, *PoCG* postcentral gyrus, *OFC* orbitofrontal cortex, *IPL* inferior parietal lobule, *SFG* superior frontal gyrus, *TIN* tinnitus, *NH* normal hearing, *HL* hearing loss, *PAC* primary auditory cortex, *DAN* dorsal attention network, *SMG* supramarginal gyrus

## Discussion

This scoping review identified 29 primary research studies that investigated resting-state networks in tinnitus patients. The majority of these studies (*n* = 26) found that resting-state networks as measured with fMRI were altered in tinnitus patients when compared to controls. Alterations were found in a variety of resting-state networks, most notably the auditory network (*n* = 19), the default mode network (*n* = 17), visual network (*n* = 14), the attention networks: dorsal attention network (*n* = 7), ventral attention network (*n* = 1), attention/executive control network (*n* = 9), and the limbic system (*n* = 8). It is important to note that the frequency of these findings depends largely on the chosen region of interest in the papers. The next paragraphs present a narrative discussion of the findings for these networks.

### Network Changes in Tinnitus

#### Auditory Network in Tinnitus

Tinnitus is an auditory perception, leading to the hypothesis that the auditory network in tinnitus patients is altered compared to controls. To investigate auditory network changes in tinnitus, several studies placed seeds in primary auditory cortex (PAC) or Heschl’s gyrus (HG), or lower on the auditory pathway.

**Fig. 3:**
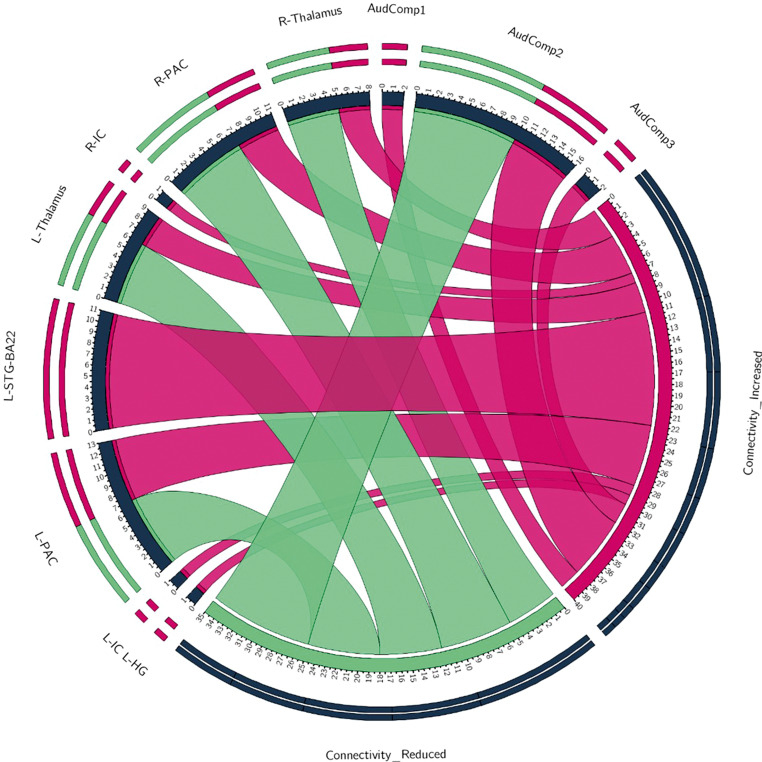
Findings of increased or decreased auditory connectivity in tinnitus patients compared to controls. Labels reflect ROIs used in the papers. Red indicates increased connectivity, and green indicates decreased connectivity. The thickness of the ribbons reflects the number of identified increased or decreased connections for that region. *L‑HG* Left Heschl’s gyrus, *L‑IC* Left Inferior Colliculus, *L‑PAC* Left Primary Auditory Cortex, *L‑STG-BA22* Left Superior Temporal Gyrus (BA22), *R‑IC* Right Inferior Colliculus, *R‑PAC* Right Primary Auditory Cortex, *AudComp1* Auditory Component consisting of Bilateral Auditory Cortex and Bilateral Non-Primary Auditory Cortex, *AudComp2* Bilateral Transverse Temporal Gyrus, Bilateral Superior Temporal Gyrus and Bilateral Insula, *AudComp3* Auditory Component consisting of Bilateral Primary Auditory Cortex

Fig. 3 presents the findings of SCA studies that used auditory regions of interest in their analysis. There were more findings of increased functional connectivity (FC) using auditory seeds than there were decreased findings but as Fig. 3 shows results were very mixed. For bilateral thalamus and PAC, both increased and decreased connections were found in tinnitus compared to controls.

Berlot et al. [7] used a high resolution 7 Tesla MRI scanner to investigate frequency specific responses in subcortical regions of the auditory pathway, such as the inferior colliculus (IC) and the medial geniculate body (MGB) in 6 tinnitus patients compared to 6 hearing-matched controls. They first used task-based fMRI to build tonotopic maps, but they did not find any differences in tonotopic organization between tinnitus patients and controls. They did not find evidence for an overrepresentation of tinnitus pitch at any level of the auditory hierarchy either. Placing seeds in PAC, they did find decreased FC with non-primary auditory cortex. They also found decreased FC between PAC and the MGB. These reductions were seen not only in voxels responsive to the tinnitus frequency, but also in control voxels.

Zhang et al. [80] investigated thalamocortical FC in tinnitus patients with normal hearing (*n* = 31) compared to matched controls (*n* = 33). They placed seeds in bilateral thalamus and found decreased FC between right thalamus and left superior temporal gyrus (STG) or Brodmann Area 42 (BA 42), which is part of non-primary auditory cortex. This finding was correlated with tinnitus duration (r = −0.454, *p* = 0.017). They also found decreased FC between left thalamus and left MTG or BA 21, which is also part of non-primary auditory cortex, which was correlated with tinnitus severity score (r = −0.482, *p* = 0.011). The observed reduced coupling between thalamus and auditory areas is in line with findings from Berlot et al. [7].

Theories of increased gain involve the MGB, of which two models of tinnitus pathology place a central role on MGB: thalamocortical dysrhythmia (TCD) and the frontostriatal gating hypothesis. The TCD hypothesis suggests hyperpolarization of the MGB results in an MGB firing mode-switch [49]. The frontostriatal gating hypothesis [57] suggests tinnitus arises due to a breakdown of limbic-auditory interactions at the level of the thalamus, or more specifically from a thalamic reticular nucleus based release of MGB inhibition. Based on these models, one would intuitively expect increased PAC-MGB connectivity, rather than decreased connectivity, as was observed in Berlot et al. [7].

Several studies have found increased auditory connectivity in tinnitus. Hinkley et al. [40] found increased FC between bilateral PAC and non-primary auditory cortex, as did Cai et al. [13]. Job et al. [44] placed seeds in bilateral HG, bilateral IC, and bilateral MGB. They did not observe any altered connectivity with the MGB in patients with non-bothersome tinnitus following acoustic trauma (*n* = 19) compared to controls (*n* = 19), as opposed to Berlot et al. [7]; however, they did observe increased FC between left HG and posterior cingulate cortex (PCC), an important region of the DMN (also see Sect. Default Mode Network in Tinnitus). Next to that, they observed increased FC between bilateral IC and right superior parietal lobule (SPL). The IC is known for its role in auditory integration, and the SPL is thought to be involved with cognitive control, so one explanation for a disturbed link between IC and SPL could be the difficulty of filtering out the tinnitus signal.

Also, the four studies using ALFF all found increased ALFF values in auditory regions [13, 17, 21, 36], indicating stronger intensity of regional neuronal activity in AC in tinnitus patients.

In summary, altered FC as well as increased ALFF has been found along the auditory pathway in tinnitus patients compared to controls; however, there is a dichotomy between studies finding increased FC and studies finding decreased FC along the auditory pathway. Potentially, this dichotomy could be explained by tinnitus heterogeneity, as these groups had differences with respect to the presence of hearing loss, the laterality of tinnitus, the duration of tinnitus, and tinnitus severity. Alternatively, as studies were small-scale, the contradictory results could also reflect false positives.

#### Default Mode Network in Tinnitus

The DMN has been described to consist of ventral medial prefrontal cortex (mPFC), PCC, IPL, lateral temporal cortex, dorsal medial prefrontal cortex, and the hippocampal formation. It is active when individuals are not performing any external task but is engaged in internal cognitive processes, such as mind-wandering or thinking about the future [11]. As tinnitus is mainly experienced at rest, which is when the DMN is most active, it has been hypothesized that aberrant functioning of the DMN may be involved with tinnitus pathophysiology. The continuous awareness of a sound might put the tinnitus patient in a task state, therefore disrupting the DMN. Aberrant connectivity of the DMN was found in several studies included in this review, but studies do not agree on whether the connectivity is increased or decreased.

The precuneus is a hub of the DMN [70] which was implicated in several studies in this review. Chen et al. [15] and Chen et al. [16] found increased FC between ACC and left precuneus, which was correlated with tinnitus severity (r = 0.507, *p* = 0.008) and tinnitus duration (r = 0.451, *p* = 0.007). Chen et al. [15] also found increased FC between PCC and right mPFC, both regions within the DMN, which was correlated with tinnitus distress (r = 0.411, *p* = 0.014). Another study by the same group [22] again found increased FC between PCC and right mPFC, this time correlated with poorer performance on a cognitive test (trail making test B, r = 0.474, *P* = 0.008), suggesting increased FC within the DMN in tinnitus patients could be related to executive dysfunction.

Zhang et al. [80] found increased FC between right thalamus and left PCC but did not observe any correlations with tinnitus characteristics. Finally, Job et al. [44] found increased FC between left HG and PCC in a group of non-bothersome tinnitus patients.

As opposed to these studies that found increased FC with DMN, Schmidt et al. [64] found decreased FC between precuneus and the rest of the DMN. They placed seeds in bilateral PAC, in the DMN, and in the DAN, and compared connectivity across tinnitus subgroups of mild, recent tinnitus (*n* = 13), mild, long-term tinnitus (*n* = 29), and bothersome long-term tinnitus (*n* = 15). They also included a normal hearing control group (*n* = 15) and a hearing loss-matched control group (*n* = 13). No differences were found when comparing the mild and bothersome tinnitus groups; however, when comparing tinnitus group to controls, or long-term tinnitus to recent tinnitus, they found decreased FC between seeds in the DMN and the precuneus, as well as increased FC between seeds in the DAN and the precuneus. This opposed pattern of increased/decreased connectivity of precuneus with DAN and DMN, respectively, could be related to the anticorrelation observed between the DAN and DMN in healthy subjects at rest, as the DAN is seen as a “task-positive” network and the DMN as a “task-negative” network [30].

Considering the evidence above, it is unclear what aberrant connectivity of the DMN in tinnitus reflects: it could be the awareness of a constant sound which disrupts the resting-state, however, this is difficult to reconcile with findings of increased FC within the DMN, which were the most common.

#### Attention Networks in Tinnitus

This paragraph discusses findings relevant to attention networks, including the DAN, VAN and executive control networks. There is some confusion around the nomenclature of attention networks [33] as mentioned in the introduction, thus it was chosen to discuss them together. Van den Heuvel and Hulshoff Pol [72], in their review of resting-state fMRI, present findings in support of two lateralized frontoparietal networks consisting of left and right superior parietal and superior frontal regions. These two networks are thought to be involved with processing of attention and memory. A third frontal network consisting of bilateral medial frontal cortex involved with executive control is also reported.

Other resting-state studies have discussed attention networks as consisting of a ventral and a dorsal stream also in frontoparietal regions [29]. The dorsal attention network is bilateral and consists of intraparietal sulcus (IPS) and the frontal eye fields (FEF). The ventral attention network is right-lateralized and involves right temporal-parietal junction (TPJ) and right ventral frontal cortex. In tinnitus studies, some papers have used the frontoparietal definition for attention networks, whereas other papers used the dorsal/ventral distinction. Many papers have also distinguished a frontal executive control or attention control network.

Job et al. [44] defined a seed at the node of the frontoparietal network based on the Human Connectome Project (www.humanconnectome.org). They found increased FC between this seed and the right middle frontal gyrus (MFG), thought to be involved with cognitive control, in patients with non-bothersome tinnitus compared to controls. Job et al. [44] suggested that this could be a reflection of the cognitive load tinnitus places on the sufferer either in maintaining tinnitus awareness constantly or in trying to filter out the tinnitus percept. They also placed a seed in the DAN, but they did not find any changes in FC with this seed, possibly due to the non-bothersome nature of the tinnitus in their subjects.

Burton et al. [12] on the other hand investigated a cohort of 17 bothersome tinnitus patients. They placed seeds in 6 networks, 1 being the attention control network (MFG, anterior insula (AI) and inferior frontal gyrus (IFG)). Compared to controls, they exhibited increased FC between left IFG and right AI. The alteration in this network could reflect the increase in cognitive resources required in bothersome tinnitus because of the ongoing effort of ignoring the tinnitus. Seeds were also selected in the DAN and VAN but no altered connectivity was found. The controls were not matched to the tinnitus group on hearing thresholds, and therefore it cannot be ruled out that the presence of high-frequency hearing loss in the tinnitus group is associated with the findings rather than the tinnitus itself.

Chen et al. [23] also found increased FC within the attention control network. They chose their seed regions based on ReHo analysis, which showed increased ReHo values in bilateral AI, amongst other regions (see Table S1 for details). Using bilateral AI as ROIs, they found increased FC between left AI and left MFG, and right AI and right MFG in normal-hearing tinnitus patients compared to controls. These findings were positively correlated with tinnitus severity scores (r = 0.459, *p* = 0.012 and r = 0.479, *p* = 0.009, respectively). These findings suggest that increased FC within the attention control network is linked to tinnitus severity and it might reflect an increased effort to maintain attention away from the tinnitus.

An ICA study also showed altered FC in attention networks, this time in the DAN in tinnitus patients with high-frequency hearing loss (*n* = 12) compared to matched hearing loss controls (*n* = 13). Schmidt et al. [63] created two DAN components using bilateral IPS seeds for DAN_1 and bilateral FEF seeds for DAN_2. They found that DAN_2 showed increased FC with parahippocampus whereas DAN_1 showed decreased FC with right supramarginal gyrus (SMG). The authors suggested that the increased FC with parahippocampus could be a compensatory attempt to manage the tinnitus by delegating to limbic regions. Several other studies have shown associations between attention nodes and the limbic system (see Sect. Limbic System in Tinnitus).

The results presented here suggest a complex relationship between attention and emotion systems in tinnitus, which is likely linked to tinnitus severity; however, there is one major potential confound not addressed in the studies on attention networks, which is that tinnitus patients participating in a tinnitus brain imaging study are likely attending to some extent to their tinnitus or their auditory modality in general during the scan, whereas controls are far less likely to deploy their attention in that way. Therefore, it is difficult to say if the results reflect increased attentional state in general in the tinnitus group during the experiment, or if the changes are due to the tinnitus itself.

#### Limbic System in Tinnitus

The limbic system is one of the evolutionarily older brain systems and is involved with emotional processing. Core regions are the (para)hippocampus, amygdala, nucleus accumbens (NAc), medial prefrontal cortex (mPFC) and ACC [54]. It is thought that the limbic system is involved with emotional reactions to tinnitus [43], and the parahippocampal gyrus (PHG) establishes the auditory memory of tinnitus and therefore prevents habituation to tinnitus [74].

This review found evidence of alterations in the limbic system in chronic tinnitus patients. Chen et al. [14] investigated amygdala FC in normal hearing depressed tinnitus patients compared to non-depressed tinnitus patients and compared to controls. They found decreased FC between amygdala and superior frontal gyrus (SFG) and MFG. The FC between amygdala and SFG was also decreased for non-depressed tinnitus patients compared to controls. The prefrontal cortex is engaged with emotional processing and executive functions and the authors suggest the altered connections between amygdala and prefrontal cortex may play a role in the attribution of negative emotional reactions to tinnitus.

In another study using GCA, Chen et al. [19] investigated FC of limbic structures (amygdala and hippocampus) in tinnitus patients (*n* = 26) with normal hearing compared to controls with normal hearing (*n* = 23). They did not replicate the finding of decreased amygdala-prefrontal FC. Instead, they found increased FC from right MFG and left IFG to left amygdala, as well as increased FC from right amygdala to right MFG, and from left MFG and right IFG to right amygdala. The tinnitus patients in both studies had similar tinnitus severity scores (mean Tinnitus Handicap Questionnaire (THQ) score between 50 and 60 for all groups) and similar tinnitus duration (means between 40 and 55 months), and all tinnitus sufferers had normal audiograms, so it is unclear why amygdala-prefrontal FC was increased in one group and decreased in the other.

The frontostriatal gating model involves limbic structures [57]. The theory poses that a frontostriatal network including vmPFC, NAc and ACC evaluates the relevance and emotional value of sensory stimuli and controls the flow of information through interaction with auditory thalamic regions. Xu et al. [77] investigated the frontostriatal circuit in tinnitus patients without hearing loss (*n* = 50) compared with well-matched controls (*n* = 55). They used GCA to investigate directional connectivity with bilateral NAc. They found increased FC between NAc and regions in prefrontal cortex which was positively correlated with THQ scores (r = 0.626, *p* < 0.001) and with tinnitus duration (r = 0.599, *p* < 0.001). Therefore, the findings show alterations in the limbic system in tinnitus patients which were associated with tinnitus severity and duration.

When considering evidence for the frontostriatal gating model generated by resting-state fMRI research, it is important to separate the theory into two types of gating: one where the tinnitus signal is persistently permitted to pass through the thalamus to the cortex, which could explain how tinnitus occurs at all, compared to a limbic-driven system that decides moment-to-moment whether tinnitus enters conscious awareness or not. The nature of resting-state fMRI is such that any changes in the limbic systems in tinnitus patients might reflect this latter system of the tinnitus reaching conscious awareness rather than the “hard problem” of tinnitus generation itself.

#### Visual Network in Tinnitus

Changes in FC with visual regions were commonly found in the studies in this review. For example, Burton et al. [12] placed seeds in primary visual cortex and in primary auditory cortex in tinnitus patients with hearing loss (*n* = 17) and compared to normal hearing controls (*n* = 17). They found a phase reversal in resting-state activity between the two systems characterized by negative correlations. When BOLD signal went up in one system, it went down in the other. The authors suggest this finding could reflect inhibitory circuits between the two systems, where activation of one sensory system inhibits activation of the other, “non-relevant” sensory system. In tinnitus patients, this would mean the constant activation of the auditory modality because of the perception of a phantom sound, decreases the activation of visual regions through inhibitory circuits.

Using ICA, Maudoux et al. [50] investigated connectivity of the auditory network in tinnitus patients with mild-to-severe hearing loss (*n* = 13) compared to age-matched but not hearing-matched controls (*n* = 15). They selected 14 seed regions in an automated way to create the auditory component, and one finding was decreased connectivity with occipital cortex. They also found increased FC with precentral and postcentral gyrus. The authors suggested the links between the auditory system and visual and sensory-motor network in tinnitus patients could have something to do with clinical observations of tinnitus sufferers who are able to modify their tinnitus perception using eye movements or head and neck movements.

The studies above did not control for hearing loss, but other studies that found alterations in visual networks in tinnitus patients did. Zhang et al. [80] found decreased FC between right thalamus and left middle occipital gyrus (MOG) (visual association cortex), and decreased FC between left thalamus and bilateral calcarine cortex (primary visual) in normal-hearing tinnitus patients (*n* = 31) compared to matched controls (*n* = 33).

One study found decreased ALFF values in right MOG [17] which they attributed to the tinnitus salience decreasing spontaneous activity in the visual areas through auditory-visual connections. The same group replicated this result in Chen et al. [21] where they again found decreased ALFF values this time in bilateral MOG.

No studies regarded alterations in the visual network as a cause of tinnitus; all studies saw these alterations as effects of the tinnitus.

### Methodological Challenges

The papers included in this review all demonstrated variations in methodology and study design. The most popular analysis method was seed-based whole brain FC analysis; however, the requirement of selecting a priori regions of interest in this analysis method poses problems for reproducibility of results. Only a few studies chose the exact same ROIs to investigate, and therefore the field is lacking reproducibility. Also, some studies used an “eyes open” paradigm for the resting-state acquisition whereas others used an “eyes closed” paradigm, which could explain some of the heterogeneity in findings, as the choice of paradigm was previously shown to affect visual and auditory connectivity [1].

Another concern is the heterogeneity of tinnitus groups. Tinnitus can be characterized in a lot of different ways and therefore careful selection of participants is required. Of 29 studies 15 included only tinnitus sufferers and controls without a hearing loss visible on a standard audiogram, which makes hearing loss controlled for as a covariate; however, a concern would be that the results of these studies are not generalizable to the tinnitus community at large, as up to 80% of tinnitus patients are estimated to have hearing loss [38, 75].

Another source of variation between papers could come from fMRI data preprocessing decisions. These largely depend on the software package used. As most studies in this review used a version of statistical parametric mapping (SPM), preprocessing pipelines were similar. Some differences were found in the use of band-pass filtering to extract the resting-state data, where the frequencies that the filter was applied to ranged from 0.008–0.08 Hz to 0.01–0.1 Hz. This is common practice in rs-fMRI research, but concerns have been raised that limiting the data to this frequency band might lose valuable information, as RSNs such as the DMN have been identified in higher frequency bands as well [10].

One concern is whether FC differences should be considered a cause or an effect of tinnitus, which cannot be inferred based on correlational resting-state data. It is difficult to determine what the altered FC reflects, as tinnitus involves many different cognitive components. Alterations in the visual network are usually considered an effect of the tinnitus. It is less clear how to interpret changes in the DMN and attention networks. A concern is how many of these findings are not only not a cause of tinnitus, but also not specific to tinnitus. It is likely that many of the changes in brain states observed in resting-state fMRI are common to all chronic symptomatic conditions that can cause distress and affect attentional states (e.g. chronic pain), and are therefore not specific to tinnitus.

A potential major confound in tinnitus resting-state research is attentional deployment [65]. The instructions for subjects in an MRI scanner for the resting-state task is usually “relax and do not think of anything in particular”; however, one might expect that tinnitus patients participating in such an experiment will spend some time focussing attention on their tinnitus, whereas the controls without tinnitus have no such focussed attention.

### Future Directions

Longitudinal studies of rs-fMRI and tinnitus could in theory address the issue of making inferences about causality discussed above. The difficulty here is to find tinnitus patients with such recent onset tinnitus, as by the time most people with recent onset tinnitus are seen by the health services, it is probably already too late. An alternative would be to use non-invasive brain stimulation or residual inhibition to alter the tinnitus state and to use rs-fMRI before and after the intervention.

Future research could also combine rs-fMRI with methods such as diffusion tensor imaging (DTI) to see whether changes in functional connectivity are reflected in white matter tracts. Van den Heuvel et al. [73] successfully used DTI to show that RSNs reflect the underlying anatomy of white matter tracts. It would be interesting to investigate this in tinnitus, and if there is a link with tinnitus duration.

Besides these novel approaches, future studies should focus on replicating previous findings by selecting the same ROIs and using the same methodology, to increase replicability of findings in the field. These studies should exclude participants with a history of neurological or psychiatric disorders, as this is known to affect functional connectivity. This review did not exclude studies that did not explicitly state whether they included or excluded these participants, but rather just lists each study’s exclusion criteria in supplemental table S1, which is a shortcoming of this review.

Future studies could also evaluate differences within the tinnitus populations, such as laterality, cause, pitch, presence of hearing loss, and duration. They should also try to control for differences in attentional deployment in the scanner between tinnitus patients and controls. A first step towards this could be to ask participants after their scan what they were thinking about, and if they were aware of their tinnitus during the scan. It is likely that some people can still hear their tinnitus in the MRI scanner and others cannot, depending on the maskability of their tinnitus. Controlling for sound stimulation is a known limitation of fMRI studies in the field, which future studies might want to address by comparing a traditional resting-state paradigm to a task such as “actively try to listen to your tinnitus”.

Future studies should also be more hypothesis-driven to avoid post hoc interpretations of findings. They should aim to prove or disprove established tinnitus theoretical models. Next to providing crucial insights into the neurophysiology of tinnitus, this direction of research outlined above could potentially lead to the identification of a biomarker for tinnitus. This could then be used in the development of new management strategies, or as an objective tool to track efficacy of interventions.

### Consultation Stage

The final stage of the scoping review framework is to conduct a consultation stage to gather insight from experts in the field. Six experts were invited to read the manuscript before submission and give their opinion about the findings and potential future directions. Three questions were asked:

1. Do the findings in the scoping review overlap with the impression of the field you currently hold?
2. What do you think the future direction should be for tinnitus and brain imaging/fMRI research?
3. Are there any important points worth addressing in the review that you think we missed?

Five experts replied with their thoughts. Their overall opinion was the findings aligned with their impression of the field. Several experts noted the problem of multiple comparisons in the resting-state fMRI literature. Seed-based functional connectivity studies often test a large number of different ROIs, and often it is unclear how correction for multiple comparisons was applied as there is no standard in the field on how to implement this. Therefore, the field likely suffers from false positives, which could explain why findings are divergent and sometimes directly opposing.

The need to focus on how this research could help patient care in the future was also pointed out. In order to move forward on this, tinnitus subtyping along with controlling carefully for confounds will be necessary. Also, future studies should focus on the “hard problem” of tinnitus, which is how it is generated in the first place, rather than secondary effects of the tinnitus. The complexity here is that the tinnitus generation itself is likely a very subtle alteration, which has smaller impact on distributed brain activity than its far-reaching consequences on attention and distress, which are reflected in the significant correlations between tinnitus distress/severity scores and tinnitus duration with resting-state patterns.

## Conclusion

This scoping review included 29 primary research papers investigating resting-state functional connectivity in tinnitus patients. Alterations were found in widely distributed brain networks, including the auditory network, DMN, attention networks, limbic system and visual network. The results show that tinnitus is a complex condition involving multiple overlapping networks, but it is unclear which changes are primary and which are secondary to tinnitus. Future studies should focus on replicating findings and subtyping tinnitus groups, and testing a priori hypotheses and theoretical models of tinnitus, which could potentially lead to the identification of a biomarker for tinnitus.

## Supplementary Information

Tab. S1 Resting-state fMRI studies complete data charting

## Abbreviations

ACC: Anterior cingulate cortex
AI: Anterior insula
ALFF: Amplitude of low-frequency fluctuations
BA: Brodmann’s area
BOLD: Blood-oxygen-level-dependent
DAN: Dorsal attention network
DMN: Default mode network
FMRI: Functional magnetic resonance imaging
FC: Functional connectivity
FEF: Frontal eye fields
FP: Frontoparietal
FWHM: Full width at half maximum
GE-EPI: Gradient-echo echo-planar imaging
GCA: Granger causality analysis
HG: Heschl’s gyrus
HL: Hearing loss
IC: Inferior colliculus
ICA: Independent component analysis
IFG: Inferior frontal gyrus
IPS: Intraparietal sulcus
M1: Primary motor cortex
MFG: Middle frontal gyrus
MGB: Medial geniculate body
MOG: Middle occipital gyrus
MPFC: Medial prefrontal cortex
MTG: Middle temporal gyrus
NAc: Nucleus accumbens
NH: Normal hearing
OFC: Orbitofrontal cortex
PAC: Primary auditory cortex
PCC: Posterior cingulate cortex
ReHo: Regional homogeneity
ROI: Region of interest
Rs-fMRI: Resting-state functional magnetic resonance imaging
RSN: Resting-state network
SCA: Seed-based correlation analysis
SFC: Superior frontal cortex
SFG: Superior frontal gyrus
SPC: Superior parietal cortex
SPM: Statistical parametric mapping
SPL: Superior parietal lobule
TCD: Thalamocortical dysrhythmia
TE: Echo time
THQ: Tinnitus handicap questionnaire
TPJ: Temporoparietal junction
TR: Repetition time
V1: Primary visual cortex
VAN: Ventral attention network
VMHC: Voxel-mirrored homotopic connectivity
VmPFC: Ventral medial prefrontal cortex

## References

[1] Agcaoglu O, Wilson TW, Wang YP, Stephen J, Calhoun VD (2019). Resting state connectivity differences in eyes open versus eyes closed conditions. Hum Brain Mapp.

[2] Andersson G, Kaldo-Sandström V, Ström L, Strömgren T (2003). Internet administration of the hospital anxiety and depression scale in a&nbsp;sample of tinnitus patients. J Psychosom Res.

[3] Arksey H, O’Malley L (2005). Scoping studies: towards a&nbsp;methodological framework. Int J Soc Res Methodol.

[4] Attwell D, Iadecola C (2002). The neural basis of functional brain imaging signals. Trends Neurosci.

[5] Baguley D, Andersson G, McFerran D, McKenna L (2012). Tinnitus: a multidisciplinary approach.

[6] Beckmann CF, DeLuca M, Devlin JT, Smith SM (2005). Investigations into resting-state connectivity using independent component analysis. Philos Trans R Soc Lond B Biol Sci.

[7] Berlot E, Arts R, Smit J, George E, Gulban OF, Moerel M, Stokroos Robert, Formisano Elia, De Martino F (2020). A 7 Tesla fMRI investigation of human tinnitus percept in cortical and subcortical auditory areas. Neuroimage Clin.

[8] Biswal B, Yetkin ZF, Haughton VM, Hyde JS (1995). Functional connectivity in the motor cortex of resting human brain using echo-planar mri. Magn Reson Med.

[9] Biswal BB, Kylen JV, Hyde JS (1997). Simultaneous assessment of flow and BOLD signals in resting-state functional connectivity maps. Nmr Biomed.

[10] Boubela R, Kalcher K, Huf W, Kronnerwetter C, Filzmoser P, Moser E (2013). Beyond noise: using temporal ICA to extract meaningful information from high-frequency fMRI signal fluctuations during rest. Front Hum Neurosci.

[11] Buckner RL, Andrews-Hanna JR, Schacter DL (2008). The brain’s default network: anatomy, function, and relevance to disease. Ann N Y Acad Sci.

[12] Burton H, Wineland A, Bhattacharya M, Nicklaus J, Garcia KS, Piccirillo JF (2012). Altered networks in bothersome tinnitus: a&nbsp;functional connectivity study. BMC Neurosci.

[13] Cai W-W, Li Z-C, Yang Q-T, Zhang T (2019). Abnormal spontaneous neural activity of the central auditory system changes the functional connectivity in the tinnitus brain: a resting-state functional MRI study. Front Neurosci.

[14] Chen Y-C, Bo F, Xia W, Liu S, Wang P, Su W, Xu J-J, Xiong Z, Yin X (2017). Amygdala functional disconnection with the prefrontal-cingulate-temporal circuit in chronic tinnitus patients with depressive mood. Prog Neuro Psychopharmacol Biol Psychiatr.

[15] Chen Y-C, Chen H, Bo F, Xu J-J, Deng Y, Lv H, Cai Y, Xia W, Yin X, Gu J-P, Lu G (2018). Tinnitus distress is associated with enhanced resting-state functional connectivity within the default mode network. Neuropsyhicatric Dis Treat.

[16] Chen Y-C, Liu S, Lv H, Bo F, Feng Y, Chen H, Xu J-J, Yin X, Wang S, Gu J-P (2018). Abnormal resting-state functional connectivity of the anterior cingulate cortex in unilateral chronic tinnitus patients. Front Neurosci.

[17] Chen Y-C, Zhang J, Li X-W, Xia W, Feng X, Gao B, Ju S-H, Wang J, Salvi R, Teng G-J (2014). Aberrant spontaneous brain activity in chronic tinnitus patients revealed by resting-state functional MRI. Neuroimage Clin.

[18] Chen YC, Feng Y, Yin X (2016). Disrupted brain functional network architecture in chronic tinnitus patients. Neuroradiology.

[19] Chen YC, Xia W, Chen H, Feng Y, Xu JJ, Gu JP, Salvi R, Yin X (2017). Tinnitus distress is linked to enhanced resting-state functional connectivity from the limbic system to the auditory cortex. Hum Brain Mapp.

[20] Chen YC, Xia W, Feng Y, Li X, Zhang J, Feng X, Wang CX, Cai Y, Wang J, Salvi R, Teng GJ (2015). Altered interhemispheric functional coordination in chronic tinnitus patients. Biomed Res Int.

[21] Chen YC, Xia W, Luo B, Muthaiah VPK, Xiong Z, Zhang J, Wang J, Salvi R, Teng GJ (2015). Frequency-specific alternations in the amplitude of low-frequency fluctuations in chronic tinnitus. Front Neural Circuits.

[22] Chen YC, Zhang H, Kong YY, Lv H, Cai YX, Chen HY, Feng Y, Yin XD (2018). Alterations of the default mode network and cognitive impairment in patients with unilateral chronic tinnitus. Quant Imaging Med Surg.

[23] Chen YC, Zhang J, Li XW, Xia W, Feng X, Qian C, Teng GJ (2015). Altered intra- and interregional synchronization in resting-state cerebral networks associated with chronic tinnitus. Neural Plast.

[24] Cordes D, Haughton VM, Arfanakis K, Wendt GJ, Turski PA, Moritz CH, Quigley MA, Meyerand ME (2000). Mapping functionally related regions of brain with functional connectivity MR imaging. Am J Neuroradiol.

[25] Damoiseaux JS, Rombouts SA, Barkhof F, Scheltens P, Stam CJ, Smith SM, Beckmann CF (2006). Consistent resting-state networks across healthy subjects. Proc Natl Acad Sci USA.

[26] Davies J, Gander PE, Andrews M, Hall DA (2014). Auditory network connectivity in tinnitus patients: A resting-state fMRI study. Int J Article Audiol.

[27] Esmaili AA, Renton J (2018). A review of tinnitus. Aust J Gen Pract.

[28] Feng Y, Chen YC, Lv H, Xia WQ, Mao CN, Bo F, Yin XD (2018). Increased resting-state cerebellar-cerebral functional connectivity underlying chronic Tinnitus. Front Aging Neurosci.

[29] Fox MD, Corbetta M, Snyder AZ, Vincent JL, Raichle ME (2006). Spontaneous neuronal activity distinguishes human dorsal and ventral attention systems. Proc Natl Acad Sci USA.

[30] Fox MD, Snyder AZ, Vincent JL, Corbetta M, Van Essen DC, Raichle ME (2005). The human brain is intrinsically organized into dynamic, anticorrelated functional networks. Proc Natl Acad Sci USA.

[31] Gentil A, Deverdun J, de Champfleur NM, Puel J-L, Le Bars E, Venail F (2019). Alterations in regional homogeneity in patients with unilateral chronic Tinnitus. Trends Hear.

[32] Granger CWJ (1969). Investigating causal relations by econometric models and cross-spectral methods. Econometrica.

[33] Gratton C, Sun H, Petersen SE (2018). Control networks and hubs. Psychophysiology.

[34] Greicius MD, Flores BH, Menon V, Glover GH, Solvason HB, Kenna H, Reiss AL, Schatzberg AF (2007). Resting-State Functional Connectivity in Major Depression: Abnormally Increased Contributions from Subgenual Cingulate Cortex and Thalamus. Biol Psychiat.

[35] Greicius MD, Srivastava G, Reiss AL, Menon V (2004). Default-mode network activity distinguishes alzheimer’s disease from healthy aging: evidence from functional MRI. Proc Natl Acad Sci USA.

[36] Han Q, Zhang Y, Liu DH, Wang Y, Feng YJ, Yin XT, Wang J (2018). Disrupted local neural activity and functional connectivity in subjective tinnitus patients: evidence from resting-state fMRI study. Neuroradiology.

[37] Henderson-Sabes J, Shang Y, Perez PL, Chang JL, Pross SE, Findlay AM, Mizuiri D, Hinkley LB, Nagarajan SS, Cheung SW (2019). Corticostriatal functional connectivity of bothersome tinnitus in single-sided deafness. Sci Rep.

[38] Henry JA, Dennis KC, Schechter MA (2005). General review of Tinnitus: prevalence, mechanisms, effects, and management. J. Speech Lang. Hear. Res..

[39] Henry JA, Reavis KM, Griest SE, Thielman EJ, Theodoroff SM, Grush LD, Carlson KF (2020). Tinnitus: an epidemiologic perspective. Otolaryngol Clin North Am.

[40] Hinkley LB, Mizuiri D, Hong O, Nagarajan SS, Cheung SW (2015). Increased striatal functional connectivity with auditory cortex in tinnitus. Front Hum Neurosci.

[41] Hofmann E, Behr R, Neumann-Haefelin T, Schwager K (2013). Pulsatile tinnitus: imaging and differential diagnosis. Dtsch Arztebl Int.

[42] Husain FT, Schmidt SA (2014). Using resting state functional connectivity to unravel networks of tinnitus. Hear Res.

[43] Jastreboff PJ, Jastreboff MM (2000). Tinnitus Retraining Therapy (TRT) as a&nbsp;method for treatment of tinnitus and hyperacusis patients. J Am Acad Audiol.

[44] Job A, Jaroszynski C, Kavounoudias A, Jaillard A, Delon-Martin C (2020). Functional connectivity in chronic nonbothersome Tinnitus following acoustic trauma: a seed-based resting-state functional magnetic resonance imaging study. Brain Connect.

[45] Jouan-Rimbaud Bouveresse D, Moya-González A, Ammari F, Rutledge DN (2012). Two novel methods for the determination of the number of components in independent components analysis models. Chemometr Intell Lab Syst.

[46] Langers DR, de Kleine E, van Dijk P (2012). Tinnitus does not require macroscopic tonotopic map reorganization. Front Syst Neurosci.

[47] Lee MH, Smyser CD, Shimony JS (2013). Resting-state fMRI: a&nbsp;review of methods and clinical applications. AJNR Am J Neuroradiol.

[48] Lee MH, Solowski N, Wineland A, Okuyemi O, Nicklaus J, Kallogjeri D, Piccirillo JF, Burton H (2012). Functional connectivity during modulation of Tinnitus with orofacial maneuvers. Otolaryngol Head Neck Surg.

[49] Llinás RR, Ribary U, Jeanmonod D, Kronberg E, Mitra PP (1999). Thalamocortical dysrhythmia: a neurological and neuropsychiatric syndrome characterized by magnetoencephalography. Proc Natl Acad Sci USA.

[50] Maudoux A, Lefebvre P, Cabay JE, Demertzi A, Vanhaudenhuyse A, Laureys S, Soddu A (2012). Auditory resting-state network connectivity in tinnitus: a&nbsp;functional MRI study. PLoS ONE.

[51] Mays N, Roberts E, Popay J, Fulop N, Allen P, Clarke A, Black N (2001). Synthesising research evidence. Studying the organisation and delivery of health services: research methods.

[52] Meszlényi RJ, Hermann P, Buza K, Gál V, Vidnyánszky Z (2017). Resting state fMRI functional connectivity analysis using dynamic time warping. Front Neurosci.

[53] Minami SB, Oishi N, Watabe T, Uno K, Ogawa K (2018). Auditory related resting state fMRI functional connectivity in Tinnitus patients: Tinnitus diagnosis performance. Otol Neurotol.

[54] Morgane PJ, Galler JR, Mokler DJ (2005). A review of systems and networks of the limbic forebrain/limbic midbrain. Prog Neurobiol.

[55] Ocran E. Cerebral cortex. 2021. https://www.kenhub.com/en/library/anatomy/cerebral-cortex. Accessed 15 Dec 2021.

[56] Ogawa S, Lee TM, Kay AR, Tank DW (1990). Brain magnetic resonance imaging with contrast dependent on blood oxygenation. Proc Natl Acad Sci USA.

[57] Rauschecker JP, Leaver AM, Mühlau M (2010). Tuning out the noise: limbic-auditory interactions in Tinnitus. Neuron.

[58] Rombouts SARB, Damoiseaux JS, Goekoop R, Barkhof F, Scheltens P, Smith SM, Beckmann CF (2009). Model-free group analysis shows altered BOLD FMRI networks in dementia. Hum Brain Mapp.

[59] Roy CS, Sherrington CS (1890). On the regulation of the blood-supply of the brain. J Physiol.

[60] Schaette R, Kempter R (2006). Development of tinnitus-related neuronal hyperactivity through homeostatic plasticity after hearing loss: a&nbsp;computational model. Eur J Neurosci.

[61] Schaette R, McAlpine D (2011). Tinnitus with a&nbsp;normal audiogram: physiological evidence for hidden hearing loss and computational model. J Neurosci.

[62] Schecklmann M, Vielsmeier V, Steffens T, Landgrebe M, Langguth B, Kleinjung T (2012). Relationship between Audiometric slope and tinnitus pitch in tinnitus patients: insights into the mechanisms of tinnitus generation. Plos One.

[63] Schmidt SA, Akrofi K, Carpenter-Thompson JR, Husain FT (2013). Default mode, dorsal attention and auditory resting state networks exhibit differential functional connectivity in tinnitus and hearing loss. PLoS ONE.

[64] Schmidt SA, Carpenter-Thompson J, Husain FT (2017). Connectivity of precuneus to the default mode and dorsal attention networks: A possible invariant marker of long-term tinnitus. Neuroimage Clin.

[65] Sedley W (2019). Tinnitus: does gain explain?. Neuroscience.

[66] Seydell-Greenwald A, Leaver AM, Turesky TK, Morgan S, Kim HJ, Rauschecker JP (2012). Functional MRI evidence for a&nbsp;role of ventral prefrontal cortex in tinnitus. Brain Res.

[67] Sindhusake D, Mitchell P, Newall P, Golding M, Rochtchina E, Rubin G (2003). Prevalence and characteristics of tinnitus in older adults: The Blue Mountains Hearing Study. Int J Audiol.

[68] Sorg C, Göttler J, Zimmer C (2015). Imaging neurodegeneration: steps toward brain network-based pathophysiology and its potential for multi-modal imaging diagnostics. Clin Neuroradiol.

[69] Trevis KJ, Tailby C, Grayden DB, McLachlan NM, Jackson GD, Wilson SJ (2017). Identification of a&nbsp;neurocognitive mechanism underpinning awareness of chronic Tinnitus. Sci Rep.

[70] Utevsky AV, Smith DV, Huettel SA (2014). Precuneus is a&nbsp;functional core of the default-mode network. J Neurosci.

[71] Van den Heuvel M, Mandl R, Pol HH (2008). Normalized cut group clustering of resting-state fMRI data. Plos One.

[72] Van den Heuvel MP, Pol HHE (2010). Exploring the brain network: a&nbsp;review on resting-state fMRI functional connectivity. Eur Neuropsychopharmacol.

[73] Van den Heuvel MP, Mandl RCW, Kahn RS, Pol HEH (2009). Functionally linked resting-state networks reflect the underlying structural connectivity architecture of the human brain. Hum Brain Mapp.

[74] Vanneste S, van de Heyning P, De Ridder D (2011). The neural network of phantom sound changes over time: a&nbsp;comparison between recent-onset and chronic tinnitus patients. Eur J Neurosci.

[75] Vernon JA, Meikle MB, Tyler RS (2000). Tinnitus masking. Tinnitus handbook.

[76] Wineland AM, Burton H, Piccirillo J (2012). Functional connectivity networks in nonbothersome Tinnitus. Otolaryngol Neck Surg.

[77] Xu JJ, Cui JL, Feng Y, Yong W, Chen HY, Chen YC, Yin XD, Wu YQ (2019). Chronic Tinnitus exhibits bidirectional functional dysconnectivity in frontostriatal circuit. Front Neurosci.

[78] Yang S, Weiner BD, Zhang LS, Cho S-J, Bao S (2011). Homeostatic plasticity drives tinnitus perception in an animal model. Proc Natl Acad Sci USA.

[79] Zang Y, Jiang T, Lu Y, He Y, Tian L (2004). Regional homogeneity approach to fMRI data analysis. Neuroimage.

[80] Zhang J, Chen Y-C, Feng X, Yang M, Liu B, Qian C, Wang J, Salvi R, Teng G-J (2015). Impairments of thalamic resting-state functional connectivity in patients with chronic tinnitus. Eur J Article Radiol.

[81] Zimmerman BJ, Abraham I, Schmidt SA, Baryshnikov Y, Husain FT (2019). Dissociating tinnitus patients from healthy controls using resting-state cyclicity analysis and clustering. Netw Neurosci.

