# Supplementary material for: Resting-state Networks in Tinnitus: A Scoping Review
Source: Clin Neuroradiol. 2022 May 12;32(4):903–22. doi: 10.1007/s00062-022-01170-1 (PMC9744700; doi:10.1007/s00062-022-01170-1)
Supplement: Supplementary file 1 — Tab. S1 Resting-state fMRI studies complete data charting [file 62_2022_1170_MOESM1_ESM.docx]

| **Resting-state fMRI studies using seed-based functional connectivity analysis** | | | | | | | | | | | | |
| --- | --- | --- | --- | --- | --- | --- | --- | --- | --- | --- | --- | --- |
| **Non-directional connectivity studies** | | | | | | | | | | | | |
| **Nr** | **Study** | **Study information** | **Tinnitus group(s) information:** | **Control group(s) information:** | **Scanning information** | **Data pre-processing** | **Seed regions (ROIs)** | **Regions showing increased connectivity in tinnitus compared to controls** | **Regions showing decreased connectivity in tinnitus compared to controls** | **Additional findings** | **Networks associated with the altered connectivity in chronic tinnitus** |  |
| 1 | Berlot et al., 2020  A 7 Tesla fMRI investigation of human tinnitus percept in cortical and subcortical auditory areas | **Aim:**  To investigate the frequency-specific processing in sub-cortical and cortical regions in tinnitus patients compared to controls  **Inclusion Criteria:**  unilateral tinnitus (2 left and 4 right), chronic (>6 months), subjective tinnitus, note: all right-handed  **Exclusion Criteria:**  Decreased sound tolerance, phonophobia, misophonia, cochlear dead regions (tested by threshold-equalising noise (TEN) test, hearing loss in pure tone over 50dB HL for both ears, a diff in average hearing threshold of more than 10dB b/w right and left ear, history of neurological / psychiatric disorders  **Study Location:**  Maastricht | **Sample Size:**  n=-6  **Sex:**  4 F/2 M  **Age:**  45.4 ± 12.4  **Tinnitus Duration:**  Not given  **Tinnitus Lateralisation:**  All unilateral 2 L/4 R  **Tinnitus Severity:**  Not given  **Tinnitus Pitch:**  Patients experienced tinnitus pitch at the following frequencies: 205, 2660, 4470, 5600, 6000 and 8000 Hz.  **Tinnitus Sound:**  Not given  **Hearing Information:**  Participants were excluded if hearing loss in pure tone over 50dB HL for both ears, a diff in average hearing threshold of more than 10dB b/w right and left ear  **Reported Comorbidities:**  None | **Sample size:**  n=-6  **Hearing:** no or mild symmetrical HL  **Sex:**  4 F/2 M  **Age:**  44.8 ± 12.3  **Matched to tinnitus group for:**  Age  Sex  Hearing thresholds  Handedness | **Scanner Strength:**  7T  **Voxel Size:**  Anatomical: 0.6x0.6x0.6 mm^3^  Functional: 1.5x1.5x1.5 mm^3^  **Image Acquisition:**  TR: 2000 ms  TE: 19 ms  **Instructions in Scanner:**  Participants were asked to lie still and fixate on a white cross presented on a black background for ten minutes. | **Distortion Correction**  Geometric distortion correction (with FSL's distortion correction tool pop-up (estimating the voxels; displacement based on data collected with the opposite phase encoding polarities) temporal high-pass filtering (removing drifts of four cycles or fewer per run)  **Software Used:**  Brain voyager QX (Brian Innovations, Maastricht) + custom MATLAB scripts  **Motion Correction:**  3D motion correction (with trilinear / sinc interpolation and aligning each volume to the first volume of functional run 1)  **Slice timing Correction:**  Yes, slice-can-time correction (with sinc interpolation)  **Spatial Smoothing:**  Temporal smoothing  **Spatial Normalisation:**  Talairach space | **Auditory (n=4)**   - Primary auditory cortex - Non-primary auditory cortex - Medial geniculate body - Inferior colliculus | None found | PAC   - Non-primary auditory cortex   PAC   - Medial geniculate body | The reduced connectivity between MGB and PAC was not specific to tinnitus pitch | Auditory network |  |
| 2 | Chen et al., 2017  Amygdala functional disconnection with the prefrontal-cingulate-temporal circuit in chronic tinnitus patients with depressive mood | **Aim:**  To investigate the disrupted amygdala-cortical FC in chronic tinnitus with depressive mood using resting-state fMRI  **Inclusion Criteria:**  Chronic tinnitus (>6 months)  **Exclusion Criteria:**  Hyperacusis, Meniere's diseases, objective tinnitus, a past history of; severe alcoholism, smoking, head injury, stroke, Alzheimer's disease, Parkinson's disease, epilepsy, major depression or other neurological or psychiatric illness, major medical illness, MRI contraindications or severe visual loss.  **Study Location:**  Nanjing, China | **Sample Size:**  n=40 (Depressive: n=20; Non-depressive: n = 20)  **Sex:**  25 F/15 M (Depressive: 12 F/8 M; Non-depressive: 13 F / 7 M)  **Age:**  Depressive: 49.75 ± 11.73 years  Non-depressive: 53.60 ± 10.03 years  **Tinnitus Duration:**  Depressive: 44.10 ± 40.14 months  Non-depressive: 51.60 ± 36.49 months  **Tinnitus Lateralisation:**  Bilateral/in the head: n=13.  Unilateral: n=27 (15 L/12 R)  **Tinnitus Severity:**  Depressive: 56.78 ± 12.98  Non-depressive: 51.17 ± 13.75  **Tinnitus Pitch:**  Not given  **Tinnitus Sound:**  Not given  **Hearing Information:**  Normal hearing on PTA  **Reported Comorbidities:**  Depression based on Self-Rating Depression Scale SDS | **Sample size:**  n=23  **Hearing:** normal on PTA  **Sex:**  13 F/10 M  **Age:**  47.13 ± 12.17 years  **Matched to tinnitus group for:**  Age  Sex  Hearing thresholds  Years of education SAS/SDS scores | **Scanner Strength:**  3T  **Voxel Size:**  Anatomical: 1x1x1 mm^3^  Resting state: 3.75x3.75x4.0 mm^3^  **Image Acquisition:**  TR: 2000 ms  TE: 30 ms  **Instructions in Scanner:**  Rest quietly with eyes closed but remain awake and avoid thinking of anything in particular. | **Distortion Correction**  Detrending and filtering (0.01–0.08Hz)  **Software Used:**  REST    **Motion Correction:**  Realignment  **Slice timing Correction:**  Yes  **Spatial Smoothing:**  Yes, Gaussian kernel, 6 mm FWHM  **Spatial Normalisation:**  MNI template | **Auditory (n=0)**  **Non-auditory (n=2)**   - L amygdala - R amygdala | **All tinnitus vs. healthy controls:**  L amygdala   - L postcentral gyrus   R amygdala   - L postcentral gyrus   **Depressive tinnitus vs non-depressive tinnitus:**  L amygdala   - L postcentral gyrus   R amygdala   - R lingual gyrus - R postcentral gyrus   **Non-depressive tinnitus vs. healthy controls:**  L amygdala   - L postcentral gyrus   R amygdala   - L postcentral gyrus   **Depressive tinnitus vs. healthy controls:**  L amygdala   - L lingual gyrus   R amygdala   - R postcentral gyrus | **All tinnitus vs. healthy controls:**  L amygdala   - L superior temporal gyrus - L middle frontal gyrus - R posterior cingulate cortex   R amygdala:   - L superior temporal gyrus - R superior frontal gyrus - R middle frontal gyrus   **Depressive tinnitus vs. non-depressive tinnitus:**  L amygdala:   - L superior frontal gyrus - L middle frontal gyrus   R amygdala:   - L middle frontal gyrus - L anterior cingulate cortex   **Non-depressive tinnitus vs. healthy controls:**  L amygdala   - L superior frontal gyrus - R posterior cingulate cortex   R amygdala:   - R superior frontal gyrus   **Depressive tinnitus vs. healthy controls:**  L amygdala   - L middle temporal gyrus - L posterior cingulate cortex   R amygdala   - L superior temporal gyrus - L superior frontal gyrus - L anterior cingulate cortex | No significant correlation found between the altered amygdala FC in depressed/non-depressed tinnitus patients and Self-rating Depression Scale scores | - Prefrontal-cingulate-temporal circuit - Executive control of attention network - Default mode network - Somatosensory network - Visual network |  |
| 3 | Chen et al., 2018  Tinnitus distress is associated with enhanced resting-state functional connectivity within the default mode network | **Aim:**  To determine whether tinnitus disrupted FC patterns within the DMN as measured by using rs-fMRI.  **Inclusion Criteria:**  Chronic tinnitus (>6 months)  **Exclusion Criteria:**  pulsatile tinnitus, hyperacusis, Meniere’s diseases, or had a past history of otologic surgery, ototoxic drug therapy, noise exposure, or hearing aid use, severe smoking, stroke, alcoholism, brain injury, Parkinson’s disease, Alzheimer’s disease, epilepsy, major depression, neurological or psychiatric disorders that could affect cognitive function, major medical illness, MRI contraindications, or severe visual loss.  **Study Location:**  Nanjing Medical University, China | **Sample Size:**  n=40  **Sex:**  21 F/19 M  **Age:**  53.6 ± 12.5 years  **Tinnitus Duration:**  41.9 ± 34.5 months    **Tinnitus Lateralisation:**  All bilateral (n=40)  **Tinnitus Severity:**  THQ: 52.1 ± 15.9  **Tinnitus Pitch:**  Not given  **Tinnitus Sound:**  Not given  **Hearing Information:**  Normal hearing on PTA  **Reported Comorbidities:**  Mild depression and mild anxiety were present in some members of both the tinnitus and the control group. | **Sample size:**  n=41  **Hearing:**  Normal on PTA  **Sex:**  25 F/16 M  **Age:**  49.5 ± 10.5 years  **Matched to tinnitus group for:**  Age  Sex  Hearing thresholds  Years of education  Handedness | **Scanner Strength:**  3T  **Voxel Size:**  Anatomical: 1x1x1 mm^3^  Resting state: 3.75x3.75x4.0 mm^3^  **Image Acquisition:**  TR: 2000 ms  TE: 30 ms  **Instructions in Scanner:**  Lie quietly with eyes closed without falling asleep, do not think of anything in particular, and avoid any head motion during the scan. Ear plugs with 32 dB noise attenuation were worn. | **Distortion Correction**  Detrending and Filtering (0.01- 0.08 Hz)  **Software Used:**  Data Processing Assistant for Resting State fMRI (DPARSF) programs based on SPM12, and REST  **Motion Correction:**  Realignment  **Slice timing Correction:**  Yes  **Spatial Smoothing:**  Yes, Gaussian kernel FWHM = 6mm  **Spatial Normalisation:**  MNI Template | **Auditory (n=0)**  **Non-auditory (n=2)**   - Anterior cingulate cortex - Posterior cingulate cortex | Anterior cingulate cortex   - L precuneus   Posterior cingulate cortex   - R medial prefrontal cortex | None found | - Enhanced FC between ACC and L precuneus was positively correlated with tinnitus duration (r=0.451, p=0.007) - Enhanced FC between PCC and R mPFC was positively correlated with tinnitus distress (r=0.411, p=0.014) | Default mode network |  |
| 4 | Chen et al., 2018  Abnormal Resting-State Functional Connectivity of the Anterior Cingulate Cortex in Unilateral Chronic Tinnitus Patients | **Aim:**  To illuminate the functional connectivity (FC) network  of the ACC subregions in chronic tinnitus patients using resting state fMRI  **Inclusion Criteria:**  Unilateral chronic tinnitus (>6 months)  **Exclusion Criteria:**  Meniere’s diseases, pulsatile tinnitus, or hyperacusis, or if they had a past history of severe alcoholism, smoking, head injury, stroke, Alzheimer’s disease, Parkinson’s disease, epilepsy, major depression, or other neurological or psychiatric illness, major medical illness, MRI contraindications, and severe visual loss.  **Study Location:**  Nanjing Medical University, China | **Sample Size:**  n=31  **Sex:**  17 F/14 M    **Age:**  51.4 ± 13.3 years  **Tinnitus Duration:**  40.6 ± 35.5 months  **Tinnitus Lateralisation:**  All unilateral, left sided (n=31)  **Tinnitus Severity:**  THQ: 51.7 ± 15.8  **Tinnitus Pitch:**  Not given  **Tinnitus Sound:**  Not given  **Hearing Information:**  Normal hearing on PTA  **Reported Comorbidities:**  No depression or anxiety according to SDS/SAS scores. | **Sample size:**  n=40  **Hearing:**  Normal on PTA  **Sex:**  21 F/19 M  **Age:**  48.2 ± 14.2 years  **Matched to tinnitus group for:**  Age  Sex  Years of education Hearing thresholds  SDS & SAS scores  Brain parenchyma volume  Handedness | **Scanner Strength:**  3T  **Voxel Size:**  Anatomical: 1x1x1 mm^3^  Resting state: 3.75x3.75x4.0 mm^3^  **Image Acquisition:**  TR: 2000 ms  TE: 30 ms  **Instructions in Scanner:**  Lie quietly with eyes closed without falling asleep, do not think of anything in particular, and avoid any head motion during the scan. Ear plugs with 32 dB noise attenuation were worn. | **Distortion Correction**  Detrending and filtering (0.01–0.08Hz)  **Software Used:**  Data Processing Assistant for Resting State fMRI (DPARSF) programs based on SPM8, and REST  **Motion Correction:**  Realignment  **Slice timing Correction:**  Yes  **Spatial Smoothing:**  Gaussian kernel FWHM = 6mm  **Spatial Normalisation:**  MNI template | **Auditory (n=0)**  **Non auditory (n= 2)**   - Rostral Anterior Cingulate Cortex - Dorsal Anterior Cingulate Cortex | Rostral Anterior Cingulate Cortex   - L Precuneus - R Postcentral Gyrus - R Putamen   Dorsal Anterior Cingulate Cortex   - R Superior Temporal Gyrus - R Inferior Parietal Lobule - R Orbitofrontal Cortex - R Medial Prefrontal Gyrus | Rostral Anterior Cingulate Cortex   - L Calcarine Cortex   Dorsal Anterior Cingulate Cortex   - R Fusiform Gyrus | - Enhanced FC between rostral ACC and L precuneus was positively correlated with tinnitus severity (r=0.507, p=0.008) - Enhanced FC between dorsal ACC and r IPL was positively correlated with tinnitus severity (r=0.447, p=0.022) | - Auditory network - Default mode network - Visual network - Executive functions/frontal network   Somatosensory network |  |
| 5 | Chen et al., 2018  Alterations of the default mode network and cognitive impairment in patients with unilateral chronic tinnitus | To investigate the intrinsic functional connectivity pattern within the default mode network and its associations with cognitive impairment in tinnitus patients using a resting-state fMRI  **Inclusion Criteria:**  Right-sided chronic tinnitus (>6 months)  **Exclusion Criteria:**  Meniere’s diseases, pulsatile tinnitus, or hyperacusis, or if they had a past history of severe alcoholism, smoking, head injury, stroke, Alzheimer’s disease, Parkinson’s disease, epilepsy, major depression, or other neurological or psychiatric illness, major medical illness, MRI contraindications, and severe visual loss.  **Study Location:**  Nanjing Medical University, China | **Sample Size:**  n=35  **Sex:**  20 F/15 M  **Age:**  49.94 ± 13.73 years  **Tinnitus Duration:**  37.71 ± 34.58 months    **Tinnitus Lateralisation:**  All unilateral, right sided (n=35)  **Tinnitus Severity:**  THQ: 52.22 ± 15.08  **Tinnitus Pitch:**  Not given  **Tinnitus Sound:**  Not given  **Hearing Information:**  Normal hearing on PTA  **Reported Comorbidities:**  Mean SAS and SAD was under 50 but given the SD some participants might have classified as having mild anxiety/depression | **Sample size:**  n=50  **Hearing:**  Normal on PTA  **Sex:**  30 F/20 M  **Age:**  45.16 ± 14.35 years  **Matched to tinnitus group for:**  Age  Sex  Years of education Hearing thresholds  SDS & SAS scores  Brain parenchyma volume  Grey and white matter volume  Handedness | **Scanner Strength:**  3T  **Voxel Size:**  Anatomical: 1x1x1 mm^3^  Resting state: 3.75x3.75x4.0 mm^3^  **Image Acquisition:**  TR: 2000 ms  TE: 30 ms  **Instructions in Scanner:**  Lie quietly with eyes closed without falling asleep, do not think of anything in particular, and avoid any head motion during the scan. Ear plugs with 32 dB noise attenuation were worn. | **Distortion Correction**  Detrending and filtering (0.01–0.08Hz)  **Software Used:**  Data Processing & Analysis for (Resting-State) Brain Imaging (DPABI_ V2.3_170105)  **Motion Correction:**  Realignment  **Slice timing Correction:**  Yes  **Spatial Smoothing:**  Gaussian kernel FWHM = 6mm, then detrending, then filtering (0.01-0.08 Hz)  **Spatial Normalisation:**  MNI template | **Auditory (n=0)**  **Non auditory (n=1)**   - Posterior Cingulate Cortex | Posterior cingulate cortex   - R medial prefrontal cortex | None | Enhanced FC between the PCC and right mPFC was correlated with the poorer Trail Making Test-B scores (r=0.474, P=0.008) but not with any of the other cognitive tests or with tinnitus performance | Default mode network |  |
| 6 | Feng et al., 2018  Increased Resting-State Cerebellar-Cerebral Functional Connectivity Underlying Chronic Tinnitus | **Aim:**  To illuminate the functional  connectivity network of the cerebellar regions in chronic tinnitus patients and controls using resting state fMRI  **Inclusion Criteria:**  Chronic tinnitus >6 months  **Exclusion Criteria:**  Meniere’s disease, pulsatile tinnitus or hyperacusis, or if they had a history of severe alcoholism, smoking, head injury, stroke, Alzheimer’s disease, Parkinson’s disease, epilepsy, major depression, other neurological or psychiatric illness, major medical illnesses, MRI contraindications, and/or severe vision loss.  **Study Location:**  Nanjing Medical University, China | **Sample Size:**  n=28  **Sex:**  19 F/9 M  **Age:**  50.2 ± 12.8 years  **Tinnitus Duration:**  47.8 ± 40.0 months    **Tinnitus Lateralisation:**  Bilateral/central (n=8)  Unilateral (n=20, 13 L/7 R)  **Tinnitus Severity:**  THQ: 50.8 ± 16.3  **Tinnitus Pitch:**  Not given  **Tinnitus Sound:**  Not given  **Hearing Information:**  Normal hearing on PTA  **Reported Comorbidities:**  No depression or anxiety according to SDS/SAS. | **Sample size:**  n=29  **Hearing:**  Normal on PTA  **Sex:**  19 F/10 M  **Age:**  44.3 ± 14.6 years  **Matched to tinnitus group for:**  Age  Sex  Years of education  Hearing thresholds Brain parenchyma volume  Grey and white matter volume  Handedness | **Scanner Strength:**  3T  **Voxel Size:**  Anatomical: 1x1x1 mm^3^  Resting state: 3.75x3.75x4.0 mm^3^  **Image Acquisition:**  TR: 2000 ms  TE: 30 ms  **Instructions in Scanner:**  Lie quietly with eyes closed, do not fall asleep, do not think of anything special, and avoid head motion. Ear plugs with 32 dB noise attenuation were worn. | **Distortion Correction**  Detrending and filtering (0.01–0.08Hz)  **Software Used:**  Data Processing Assistant for Resting State fMRI (DPARSF) programs based on SPM8, and REST  **Motion Correction:**  Realignment  **Slice timing Correction:**  Yes  **Spatial Smoothing:**  Gaussian kernel FWHM = 6mm, then detrending, then filtering (0.01-0.08 Hz)  **Spatial Normalisation:**  MNI template | **Auditory (n=0)**  **Non auditory (n=9)**   - Cerebellum L Crus I - Cerebellum R Crus I - Cerebellum L Crus II - Cerebellum R Crus II - Cerebellum L Lobule VI - Cerebellum R Lobule VI - Cerebellum L Lobule VIIb - Cerebellum R Lobule VIIb - Cerebellum Vermis | L Crus I   - L parahippocampal gyrus   R Crus I   - R inferior occipital gyrus   R Crus II   - R Inferior occipital gyrus   L Lobule VIIb   - R superior temporal gyrus   R Lobule VIIb   - L Precentral Gyrus   Vermis   - R Superior temporal gyrus | None found | - The increased functional connectivity between L cerebellar Lobule VIIb and R STG was positively correlated with Tinnitus Handicap Questionnaires (THQ) scores (r = 0.577, p = 0.004). - The increased functional connectivity between the cerebellar vermis and the right STG was also associated with the THQ score (r = 0.432, p = 0.039). | - Auditory network - Limbic system - Visual network |  |
| 7 | Henderson et al., 2019  Corticostriatal functional connectivity of bothersome tinnitus in single-sided deafness | **Aim:**  To define whole-brain connectivity patterns of the caudate nucleus and auditory cortex in a single sided deaf cohort with bothersome tinnitus compared to a single sided deaf cohort with no or non-bothersome tinnitus using resting-state fMRI  **Inclusion Criteria:**  Chronic tinnitus (≥1 year), constant, non-pulsatile, normal hearing in one ear and severe or profound hearing loss in the other  **Exclusion Criteria:**  Not given  **Study Location:**  San Francisco, University of California, USA. | **Sample Size:**  n = 15  **Sex:**  6 F /9 M  **Age:**  51.1 ± 8.52 years  **Tinnitus Duration:**  All >1 year (exact duration not given)  **Tinnitus Lateralisation:**  All unilateral, in deaf ear (n=15, 7 L/8 R)  **Tinnitus Severity:**  TFI: 41.7 ± 27.89  **Tinnitus Pitch:**  Not given  **Tinnitus Sound:**  Not given  **Hearing Information:**  Non-congenital, single-sided deafness  **Reported Comorbidities:**  Some had vestibular schwannoma | **Sample size:**  n = 15  **Hearing:**  Single-sided deafness  **Sex:**  6 F /9 M  **Age:**  47.2 ± 17.43 years  **Matched to tinnitus group for:**  Age  Sex  Handedness  Hearing thresholds  Duration of deafness | **Scanner Strength:**  3T  **Voxel Size:**  Anatomical: 0.5x0.5x1.5 mm^3^  Resting state: 1.88x1.88x3.0 mm^3^  **Image Acquisition:**  TR: 2000 ms  TE: 28 ms  **Instructions in Scanner:**  Ear plugs that attenuate noise by 32 dB were worn. | **Distortion Correction**  Temporal bandpass filtering (0.008Hz-0.09Hz)  **Software Used:**  CONN Toolbox  **Motion Correction:**  Functional realignment & unwarping  **Slice timing Correction:**  N/A  **Spatial Smoothing:**  Gaussian kernel FWHM = 8mm.  **Spatial Normalisation:**  MNI template | **Auditory (n=2)**   - L Heschl’s gyrus - R Heschl’s gyrus   **Non auditory (n=2)**   - L caudate nucleus - R caudate nucleus | L Caudate Nucleus   - L Heschl’s Gyrus - R supplementary motor area | None found | Within the tinnitus group, connectivity strength between the R caudate nucleus and:   - cuneus was positively correlated with TFI subscale “relax” (R^2^=0.67, p<0.05); - superior lateral occipital cortex was positively correlated with TFI subscale “control” (R^2^=0.82, p<0.05); - anterior supramarginal gyrus was positively correlated with TFI subscale “control” (R^2^=0.78, p<0.05). | - Auditory network - Limbic system - Motor network - Default mode network - Visual network - Dorsal attention network |  |
| 8 | Hinkley et al., 2015  Increased striatal functional connectivity with auditory cortex in tinnitus | **Aim:**  To test the “striatal gating model” of tinnitus by comparing rs-fMRI patterns of the striatum and auditory regions in tinnitus patients and controls  **Inclusion Criteria:**  Chronic tinnitus (>1 year)  **Exclusion Criteria:**  Not given  **Study Location:**  University of California at San Francisco, USA | **Sample Size:**  n = 15  **Sex:**  3 F/12 M  **Age:**  53.5 ± 13 years  **Tinnitus Duration:**  Not given    **Tinnitus Lateralisation:**  Bilateral (n=9)  Unilateral (n=6 3 L/3 R)  **Tinnitus Severity:**  THI: 39 ± 20.33  **Tinnitus Pitch:**  Not given  **Tinnitus Sound:**  Not given  **Hearing Information:**  Tinnitus group: wide range from mild to severe hearing loss.  **Reported Comorbidities:**  Not given | **Sample size:**  n = 15  **Hearing:**  Normal on PTA  **Sex:**  Not given  **Age:**  57 ± 12 years  **Matched to tinnitus group for:**  Age  Sex  Not matched for hearing thresholds | **Scanner Strength:**  3T  **Voxel Size:**  Anatomical:  Slice thickness = 1mm; FOV = 256mm; matrix size = not given  Resting state: 3x3x3mm^3^  **Image Acquisition:**  TR: 2000 ms  TE: 28 ms  **Instructions in Scanner:**  Eyes closed | **Distortion Correction**  Linearly detrended and bandpass filtered (second-order Butterworth; 0.01–0.08 Hz)  **Software Used:**  SPM8  **Motion Correction:**  Realignment  **Slice timing Correction:**  No  **Spatial Smoothing:**  Gaussian kernel FWHM = 8mm.  **Spatial Normalisation:**  MNI template | **Auditory (n=2)**   - L Primary Auditory Cortex - R Primary Auditory Cortex   **Non auditory (n=6)**   - L Caudate Dorsal Striatum - R Caudate Dorsal Striatum - L Caudate Head - R Caudate Head - L Nucleus Accumbens - R Nucleus Accumbens | L Primary Auditory Cortex   - R anterior superior temporal gyrus - L middle temporal gyrus - L superior frontal gyrus - L posterior cerebellum - R parahippocampal gyrus - L Lingual Gyrus   R Primary Auditory Cortex   - L middle temporal gyrus - L superior frontal gyrus - R middle occipital gyrus - R post central gyrus   L Caudate Dorsal Striatum   - L middle temporal gyrus   R Caudate Dorsal Striatum   - R superior temporal gyrus - R middle occipital gyrus - R post central gyrus   L Caudate Head   - R Putamen - R middle frontal gyrus - R cingulate - R inferior parietal lobe   R Caudate Head   - L superior frontal gyrus - R inferior parietal lobe   R Nucleus Accumbens   - L middle temporal gyrus - L superior frontal gyrus - L posterior cerebellum - L lingual gyrus - L inferior parietal lobe | L Caudate Dorsal Striatum   - L lingual gyrus - L culmen   R Caudate Dorsal Striatum   - L lingual gyrus - R lingual gyrus   R Caudate Head   - L culmen - R lingual gyrus   L Nucleus Accumbens   - R superior temporal gyrus - R culmen - L lingual gyrus - R lingual gyrus - L inferior parietal lobe | Voxelwise correlations with THI scores in the tinnitus cohort were insignificant when corrected for multiple comparisons. | - Auditory network - Visual network - Default mode network - Dorsal attention network - Limbic system |  |
| 9 | Job et al., 2020  Functional Connectivity in Chronic Nonbothersome Tinnitus Following Acoustic Trauma: A Seed-Based Resting-State Functional Magnetic Resonance Imaging Study | **Aim:**  To investigate whole-brain functional connectivity in non-bothersome tinnitus following acoustic trauma, including the role of right parietal operculum 3 (OP3).  **Inclusion Criteria:**  Chronic non-bothersome tinnitus due to acoustic trauma (duration > 6 months)  **Exclusion Criteria:**  Not given  **Study Location:**  France | **Sample Size:**  n = 19  **Sex:**  0 F/19 M  **Age:**  42.5 ± 12 years  **Tinnitus Duration:**  12.2 ± 7.3 years    **Tinnitus Lateralisation:**  Bilateral (n=13)  Unilateral (n=6 3 L/3 R)  **Tinnitus Severity:**  THI: 16.2 ± 10.5 (slight to moderate)  **Tinnitus Pitch:**  Not given  **Tinnitus Sound:**  high-pitched whistling (n=18)  medium high-pitched sizzling (n=1)  **Hearing Information:**  hearing loss at frequencies > 4kHz  **Reported Comorbidities:**  No anxiety or depression. | **Sample size:**  n = 19  **Hearing:**  Normal on PTA  **Sex:**  0 F/19 M  **Age:**  42.5 ± 11.9 years  **Matched to tinnitus group for:**  Age  Sex  Not matched for hearing thresholds | **Scanner Strength:**  3T  **Voxel Size:**  Anatomical: 0.9x0.9x1.2 mm^3^  Resting state: 3x3x3.5mm^3^  **Image Acquisition:**  TR: 2000 ms  TE: 32 ms  **Instructions in Scanner:**  Lie with eyes open and let the mind wander without focusing on anything in particular. A gray background image with a small white cross in the center was displayed. | **Distortion Correction**  Band-pass filtering (0.008 to 0.08H)  **Software Used:**  SPM12 and CONN toolbox  **Motion Correction:**  Realignment using DARTEL  **Slice timing Correction:**  Yes  **Spatial Smoothing:**  Smoothed with a small kernel (1.5mm^3^ isotropic)  **Spatial Normalisation:**  MNI template | **Auditory (n=6)**   - L inferior colliculus - R inferior colliculus - L medial geniculate body - R medial geniculate body - L Heschl’s gyrus - R Heschl’s gyrus   **Non auditory (n=5)**   - R Operculum 3 - L Operculum 3 - Anterior to R Operculum 3 - Posterior to R Operculum 3 - Whole R Operculum 3   **Whole networks (n=7)**  Based on Human Connectome Project, seeds defined using CONN toolbox   - DMN - Visual - Sensorimotor-auditory - Saliency - Language - Frontoparietal - Dorsal attentional | L Heschl’s Gyrus   - Posterior Cingulate Cortex   L Inferior Colliculus   - R Superior Parietal Lobule   R Inferior Colliculus   - R Superior Parietal Lobule   R Operculum 3   - R Superior Frontal gyrus   Posterior to R Operculum 3   - L Superior Frontal Gyrus - L Inferior Parietal Lobule   Sensorimotor–auditory network   - R paracingulate network - R posterior middle temporal gyrus - L inferior precentral gyrus   Frontoparietal network   - R middle frontal gyrus | None found | N/A | - Auditory network - Default mode network - Sensorimotor-auditory network - Frontoparietal network |  |
| 10 | Zhang et al., 2015  Impairments of thalamic resting-state functional connectivity in patients with chronic tinnitus | **Aim:**  To compare the degree of thalamocortical functional connectivity in chronic tinnitus patients and controls using resting-state fMRI.  **Inclusion Criteria:**  Chronic tinnitus (>6 months)  **Exclusion Criteria:**  Hyperacusis, pulsatile tinnitus, Meniere’s disease, history of heavy smoking, stroke, alcoholism, epilepsy, major depression, neurological or psychiatric disorders, brain injury, Parkinson’s, Alzheimer’s, major medical illness, severe visual impairment or MRI contraindications.  **Study Location:**  Hospital Southeast University, China | **Sample Size:**  n=31  **Sex:**  13 F/18 M  **Age:**  40.8 ± 13.2) years  **Tinnitus Duration:**  42.6 ± 41.1 months    **Tinnitus Lateralisation:**  Bilateral/central (n=7)  Unilateral (n=24, 14 L/10 R)  **Tinnitus Severity:**  THQ = 41.4 ± 19.7  **Tinnitus Pitch:**  Not given  **Tinnitus Sound:**  Not given  **Hearing Information:**  Normal hearing on PTA  **Reported Comorbidities:**  No depression or anxiety according to SDS/SAS | **Sample size:**  n=33  **Hearing:**  Normal on PTA  **Sex:**  15 F /18 M  **Age:**  45.2 ± 11.9 years  **Matched to tinnitus group for:**  Age  Sex  Hearing thresholds Years of education  Handedness | **Scanner Strength:**  3T  **Voxel Size:**  Anatomical: 1x1x1 mm^3^  Resting state: 3,75x3,75x4 mm^3^  **Image Acquisition:**  TR: 2000 ms  TE: 25 ms  **Instructions in Scanner:**  Keep your eyes closed but remain awake and avoid specific thoughts. Ear plugs with 32 dB attenuation were worn. | **Distortion Correction**  Detrending and filtering (0.01–0.08 Hz)  **Software Used:**  SBM8, REST, Data Processing Assistant for Resting-State fMRI, WFU_Pickatlas software, VBM8 Toolbox  **Motion Correction:**  Realignment  **Slice timing Correction:**  Yes  **Spatial Smoothing:**  Yes, Gaussian kernel FWHM = 4mm.  **Spatial Normalisation:**  MNI template | **Auditory (n=2)**   - L Thalamus - R Thalamus   **Non auditory (n=0)** | L Thalamus   - R angular gyrus - R middle cingulate cortex - L cerebellar posterior lobe   R Thalamus   - L Posterior Cingulate Cortex - L Cerebellar Posterior Lobe - R Cerebellar Posterior Lobe | L Thalamus   - R Middle Temporal Gyrus - R Middle Orbitofrontal Cortex - L Middle Frontal Gyrus - R Precentral Gyrus - L Calcarine Cortex - R Calcarine Cortex   R Thalamus   - L Superior temporal Gyrus - L Amygdala - R Superior Frontal Gyrus - L Precentral Gyrus - L Middle Occipital Gyrus | - In tinnitus patients, the functional connectivity between the left thalamus and right MTG was negatively correlated with the THQ total score (r = −0.482, p = 0.011). - The functional connectivity between the right thalamus and left STG was negatively correlated with tinnitus duration (r = −0.454, p = 0.017). | - Auditory network - Visual network - Default mode network |  |
| 11 | Schmidt et al., 2017  Connectivity of precuneus to the default mode and dorsal attention networks: A possible invariant marker of long-term tinnitus | **Aim:**  The aim was to identify resting state functional connectivity  alterations that consistently appear across tinnitus subgroups. We examined two sources of variability in the subgroups: tinnitus severity and tinnitus duration.  **Inclusion Criteria:**  Between 30 and 70 years old.  **Exclusion Criteria:**  No hyperacusis. Neurological disorders; Meniere's disease, TMJ, depression or anxiety; chronic physical disease, currently undergoing tinnitus treatment.  **Study Location:**  Illinois, University of Illinois Urbana-Champaign, USA | M=Mild, B=Bothersome, L=Long-term, R=Recent  **Sample Size:**  MRTIN: n=13 MLTIN_1: n=12 MLTIN_2: n=17 BLTIN: n=15  **Sex:**  MRTIN: 8 F/5 M MLTIN_1: 3 F/9 M MLTIN_2: 4 F/13 M BLTIN: 7 F/8 M  **Age:**  MRTIN 48.38 (± 12.15)  MLTIN_1 55 (± 6.97) MLTIN_2 51.65 (± 11.79) BLTIN 50.07 (± 10.23)  **Tinnitus Duration:**  MRTIN: between 6 and 12 months Others: >12 months  **Tinnitus Lateralisation:**  MRTIN: bilateral (n-=3), unilateral (n=1), unknowns (n=9) MLTIN_1: bilateral (n=8), unilateral left (n=2), unknown (n=2) MLTIN_2: bilateral (n=15), unilateral left (n=1), unknown (n=1) BLTIN: bilateral (n=13), unilateral right (n=1), unknown (n=1)  **Tinnitus Severity:**  MRTIN: mild (THI: 16.46 ± 4.63) MLTIN_1: mild (THI: 8.33 ± 6.76) MLTIN_2: mild (THI: 9.41 ± 4.73) BLTIN: mild – moderate (THI: 29.47 ± 10.89)  **Tinnitus Pitch:**  Not given  **Tinnitus Sound:**  Not given  **Hearing Information:**  Mean PTAs are mild to moderate hearing loss, some participants likely had no hearing loss and others a mild/moderate one. It is unclear if HL was unilateral or bilateral  **Reported Comorbidities:**  BDI and BAI scores were all minimal or mild. | **Sample size:**  NH: n=15 HL: n=13  **Hearing:**  NH: Normal on PTA  HL: mild to moderate  **Sex:**  NH: 9 M/6 F HL: 5 M/8 F  **Age:**  NH: 53 (SD 8.73) HL: 57.62 (SD 9.39)  **Matched to tinnitus group for:**  Age  Sex  Hearing thresholds (in the case of the HL control group) | **Scanner Strength:**  3T  **Voxel Size:**  Anatomical:  1.0x1.0x1.2 mm^3^ (scanner 1 & 2)  Resting state:  3.4x3.4x4.0 mm^3^ (scanner 1) 2.5x2.5x3.0 mm^3^ (scanner 2)  **Image Acquisition:**  **TR:** 2000 ms  **TE:** 30 ms (scanner 1) and 25 ms (scanner 2)  **Instructions in Scanner:**  Subjects were instructed to lay still and fixate on a cross for the duration of the scan. They wore ear plugs and headphones. | **Distortion Correction**  None given  **Software Used:**  SPM8  **Motion Correction:**  Six-parameter rigid body transformation  **Slice timing Correction:**  Yes  **Spatial Smoothing:**  Yes, Gaussian kernel FWHM = 10mm.  **Spatial Normalisation:**  MNI Template | **Auditory (n=2)**   - L Primary Auditory Cortex - R Primary Auditory Cortex   **DMN (n=2) combined**   - Medial Prefrontal Cortex - Posterior Cingulate Cortex   **DAN (n=4) combined**   - L Posterior Intraparietal Sulcus - R Posterior Intraparietal Sulcus - L Frontal Eye Field - R Frontal Eye Field | Auditory network: None found  Dorsal attention network:   - Precuneus - Region near L precentral gyrus (unspecified) | Default mode network:   - Precuneus - Frontal medial cortex - Lateral superior occipital cortex | No differences were found in any analyses between tinnitus severity subgroups; the differences described are between controls vs. tinnitus groups or between long-term vs. short-term tinnitus groups | - Default mode network - Dorsal attention network |  |
| 12 | Wineland et al., 2012  Functional Connectivity Networks in Nonbothersome Tinnitus | **Aim:**  To assess functional connectivity in cortical networks in patients with nonbothersome tinnitus compared with a normal healthy nontinnitus control group using rs-fMRI.  **Inclusion Criteria:**  Non-bothersome, idiopathic subjective tinnitus for at least 6 months.  **Exclusion Criteria:**  Anyone with (1) an active diagnosis of any acute or chronic brain-related neurological conditions; (2) history of head trauma, seizure, or stroke; (3) a retrocochlear lesion or anatomic/structural lesion of the brain, skull base, temporal bone, or ear; or (4) active depression or anxiety disorder or who had recently began taking medications to treat depression or anxiety.  **Study Location:**  Missouri, USA | **Sample Size:**  n = 18  **Sex:**  6 F/12 M  **Age:**  Median = 54 (IQR = 52-57)  **Tinnitus Duration:**  Median = 9 years (IQR = not given)  **Tinnitus Lateralisation:**  Bilateral (n=12)  Unilateral (n=6)  **Tinnitus Severity:**  Median THI score = 8 (IQR 4-14)  **Tinnitus Pitch:**  Not given  **Tinnitus Sound:**  Ringing (14), hum (2), hissing (2), high-tension wire (4), buzzing (2), whistle (1), clear tone (2), cicadas (1), transformer noise (1), clicking (1), crickets (2),  **Hearing Information:**  From mild to severe hearing loss  **Reported Comorbidities:**  None | **Sample size:**  n = 23  **Hearing:**  Normal on PTA  **Sex:**  11 F/12 M  **Age:**  Median = 46 (IQR = 39-54)  **Matched to tinnitus group for;**  Sex  Not matched for age  Not matched for hearing thresholds | **Scanner Strength:**  3T  **Voxel Size:**  Anatomical: 1x1x1.25 mm^3^  Resting state 4x4x4 mm^3^  **Image Acquisition:**  TR: 2200 ms  TE: 27 ms  **Instructions in Scanner:**  Participants were awake, performed no task, and kept their eyes closed in a darkened room | **Distortion Correction**  Band-pass filtering to remove nuisance variables and whole-brain signal normalization to mode 1000  **Software Used:**  FreeSurfer  **Motion Correction:**  12 parameter affine transformations  **Slice timing Correction:**  Yes  **Spatial Smoothing:**  Yes, spatially smoothed (6 mm FWHM Gaussian kernel)  **Spatial Normalisation:**  Talairach standard space | 58 spherical seed regions were defined to reflect the following networks:   - Dorsal attention network - Ventral attention network - Cognitive network - Default mode network - Auditory network - Visual network - Somatosensory network | None found | None found | N/A | None found |  |
| 13 | Burton et al., 2012  Altered networks in bothersome tinnitus: a functional connectivity study | **Aim:**  To examine functional connectivity linked to the auditory system in patients with chronic bothersome tinnitus  **Inclusion Criteria:**  Bothersome tinnitus (based on THI >38)  **Exclusion Criteria:**  Hyperacusis  **Study Location:**  Missouri, USA | **Sample size:**  n = 17  **Sex:**  12 M/5 F  **Age:**  53.5 ± 3.6 years  **Tinnitus Duration:**  8.3 ± 1.9 years  **Tinnitus Lateralisation:**  Bilateral (n=11), unilateral (n=5, 4 R / 1 L)  **Tinnitus Severity:**  53.5 ± 3.6 (THI)  Moderate n=10, severe n=7  **Tinnitus Pitch:**  Not given  **Tinnitus Sound:**  Not given  **Hearing Information:**  Minimal hearing loss for 1- 3 kHz and > 40dB for 8 kHz in 12 / 17 participants  **Reported Comorbidities:**  None | **Sample size:**  n=17  **Hearing:**  Normal on PTA  **Sex:**  7 M/10 F  **Age:**   - 1. ± 3.6 years   **Matched to tinnitus group for:**  Age  Not matched for hearing thresholds | **Scanner Strength:**  3T  **Voxel Size:**  Anatomical: 1x1x1.25 mm^3^  Resting state: 4x4x4 mm^3^  **Image Acquisition:**  TR: 2200 ms,  TE: 27 ms  **Instructions in Scanner:**  Participants were awake, performed no task and kept their eyes closed in a darkened room | **Distortion Correction**  Band-pass filter for low frequencies, and to remove nuisance variables & Whole brain mean signal intensity normalized to mode 1000 across EPI runs  **Software Packages:**  Analyze (Mayo Research Foundation, Rochester, MN  **Motion Correction:**  rigid body correction for inter-frame head motion; resliced to 2mm3 by 12 parameter affine transformations  **Slice timing Correction:**  Yes  **Spatial Smoothing:**  Yes, with 6 mm FWHM Gaussian kernel  **Spatial Normalisation:**  Talairach standard space | **Auditory network (n=2)**   - R Primary Auditory Cortex - L Primary Auditory Cortex   **Visual network (n=2)**   - R Primary Visual - L Cuneus   **Somatosensory network (n=2)**   - R Postcentral Gyrus - L Parietal Operculum   **Dorsal attention network (n=5)**   - L Posterior Intraparietal Sulcus - R Posterior Intraparietal Sulcus - L Frontal Eye Fields - R Ventral Intraparietal Sulcus   **Ventral attention network (n=2)**   - R Temporoparietal Junction - R Superior Temporal Sulcus   **Attention control network (n=4)**   - R Middle Frontal Gyrus - R Anterior Insula - L Inferior Frontal Gyrus - R Inferior Frontal Gyrus | L Inferior Frontal Gyrus   - R Anterior Insula | R Primary Auditory Cortex   - Occipital Pole - L Parietal Occipital Sulcus - Calcarine Sulcus - Cuneus - Lingual Gyri   L Primary Auditory Cortex   - Occipital Pole - L Parietal Occipital Sulcus - Calcarine Sulcus - Cuneus - Lingual Gyri   R Primary Visual Cortex   - L Superior Temporal Gyrus - L Sulcal Auditory Cortex - L Rostral Insula - L Inferior Frontal Gyrus   R Anterior Insula   - L Medial Occipital Cortex - R Medial Occipital Cortex - L Lateral Occipital Cortex - R Lateral Occipital Cortex   L Inferior Frontal Gyrus   - Medial occipital cortex | N/A | - Auditory network - Visual network - Attention control network |  |
| 14 | Minami et al., 2018  Auditory Related Resting State fMRI Functional Connectivity in Tinnitus Patients: Tinnitus Diagnosis Performance | **Aim:**  To investigate functional connectivity in tinnitus patients with and without hearing loss and to design the tinnitus diagnosis performance by resting state functional magnetic resonance imaging (rs-fMRI).  **Inclusion Criteria:**  Chronic tinnitus > 6 months  **Exclusion Criteria:**  Not given  **Study Location:**  Tokyo, Japan | **Sample Size:**  With hearing loss: n=18  Without hearing loss: n=11  **Sex:**  Not given  **Age:**  With HL median = 60  Without HL median = 37 years  **Tinnitus Duration:**  Not given  **Tinnitus Lateralisation:**  Not given  **Tinnitus Severity:**  With HL: TFI = 50 (± 21)  Without HL: TFI = 39 ± 26  **Tinnitus Pitch:**  Not given  **Tinnitus Sound:**  Not given  **Hearing Information:**  Mild HL: n=12 Moderate HL n=2  Severe HL: n=4  **Reported Comorbidities:**  Not given | **Sample size:**  n = 19  **Hearing:**  Normal on PTA  **Sex:**  Not given  **Age:**  Median = 35 years  **Matched to tinnitus group for:**  Not given  Not matched for hearing thresholds | **Scanner Strength:**  1.5T  **Voxel Size:**  Anatomical: 0.72x0.72x4 mm^3^  Resting state: 3.44×3.44x4 mm^3^  **Image Acquisition:**  TR: 2500 ms  TE: 40 ms  **Instructions in Scanner:**  Subjects were asked to lie motionless with their eyes open during the rs-fMRI acquisition. | **Distortion Correction**  Band-pass filtering (0.008 - 0.09 Hz)  **Software Used:**  SPM8 & CONN toolbox  **Motion Correction:**  Realignment  **Slice timing Correction:**  Not given  **Spatial Smoothing:**  Yes, specifics not given  **Spatial Normalisation:**  Atlas not given | **Auditory (n=6)**   - Heschl’s Gyrus - Planum temporale - Planum polare - Operculum - Insular cortex - Superior temporal gyrus   **Non auditory (n=0***)* | None? / ROI names and statistics are not legible in figures | None? / ROI names and statistics are not legible in figures | Associations within auditory ROIs were weakened in tinnitus patients according to authors, but exact ROIs and statistics are not legible due to poor image quality | Auditory network |  |
| 15 | Lee et al., 2012  Functional Connectivity during Modulation of Tinnitus with Orofacial Manoeuvres | **Aim:**  To determine changes in cortical neural networks as defined by rs-fMRI during voluntary modulation of tinnitus with  orofacial manoeuvres.  **Inclusion Criteria:**  Idiopathic, subjective, unilateral or bilateral, and chronic, non-pulsatile tinnitus + reproducible, voluntary control over their tinnitus, whether through attention redirection or an orofacial manoeuvre compatible with MRI’s motion sensitivity  **Exclusion Criteria:**  History of hyperacusis, misophonia, or neurological injury or illness  **Study Location:**  Missouri, USA | **Sample Size:**  n = 16  **Sex:**  6 F/10 M  **Age:**  53.7 ± 10.4 years  **Tinnitus Duration:**  20.5 ± 18 years  **Tinnitus Lateralisation:**  Bilateral (n=10)  Unilateral (n=6 3 L/3 R)  **Tinnitus Severity:**  THI = 32.1 ± 25.6 (0- 78), loudness rating = 5.4 ± 2.4 (scale of 0-10 of loudness)  **Tinnitus Pitch:**  Not given  **Tinnitus Sound:**  Not given  **Hearing Information:**  Ranging from mild to severe HL  **Reported Comorbidities:**  Not given | No control group - participants served as their own baseline by comparing their resting-state data to their functional data during orofacial manoeuvres | **Scanner Strength:**  3T  **Voxel Size:**  Not given  **Image Acquisition:**  TR/TE: Not given  **Instructions in Scanner:**  During the first 2 scans, tinnitus patients performed their tinnitus-altering manoeuvre, and during the last 2 scans, patients remained at rest with their eyes closed. | **Distortion Correction**  Whole-brain signal intensity normalisation to a mode of 1000  Temporal band-pass filtering for frequencies <0.1 Hz  **Software Used:**  Analyze (Mayo Research Foundation, Rochester, Minnesota)  **Motion Correction:**  Realignment  **Slice timing Correction:**  Yes  **Spatial Smoothing:**  Yes, 6 mm FWHM Gaussian kernel  **Spatial Normalisation:**  Atlas derived from 12 healthy adults https://pubmed.ncbi.nlm.nih.gov/16172003/ | 58 spherical seed regions with a diameter of 10mm were determined using Talairach coordinates in the following networks:   - Default mode network - Dorsal attention network - Ventral attention network - Cognitive/control network - Auditory network - Visual network - Somatosensory network | None found | None found | N/A | None found |  |

| **Resting-state fMRI studies using seed-based functional connectivity analysis** | | | | | | | | | | | | |
| --- | --- | --- | --- | --- | --- | --- | --- | --- | --- | --- | --- | --- |
| **Directional Connectivity Studies** | | | | | | | | | | | | |
| **Nr** | **Study** | **Study information** | **Tinnitus group(s) information:** | **Control group(s) information:** | **Scanning info** | **Data pre-processing** | **Data Analysis Information** | **Regions showing increased connectivity in tinnitus compared to controls** | **Regions showing decreased connectivity in tinnitus compared to controls** | **Additional findings** | **Networks associated with the altered connectivity in chronic tinnitus** |  |
| 16 | Chen et al., 2016  Disrupted Brain Functional Network Architecture in Chronic Tinnitus Patients | **Aim:**  To identify aberrant brain network architecture involved in chronic tinnitus, through comparing the resting-state fMRI (rs-fMRI) patterns of tinnitus patients and healthy controls.  **Inclusion Criteria:**  Chronic tinnitus (>6 months)  **Exclusion Criteria:**  Hyperacusis, pulsatile tinnitus or Meniere’s diseases, or if they had a past history of severe smoking, stroke, alcoholism, brain injury, Parkinson’s disease, AD, epilepsy, major depression, neurological or psychiatric disorders that could affect cognitive function, major medical illness (e.g., anemia, thyroid dysfunction and cancer), MRI contraindications (e.g., cochlear implants, pacemakers, cerebral aneurysm clips, prosthetic valves, a history of intraocular metal fragments, and claustrophobia), or severe visual loss.  **Study Location:**  Nanjing Medical University, China | **Sample Size:**  n = 24  **Sex:**  15 F/9 M  **Age:**  50.8 ± 12.4 years  **Tinnitus Duration:**  46.5 ± 39.1 (6–120) months    **Tinnitus Lateralisation:**  Bilateral/in the head (n=8)  Unilateral (n=16, 10 L/6 R)  **Tinnitus Severity:**  THQ: 49.5 ± 15.5  **Tinnitus Pitch:**  Not given  **Tinnitus Sound:**  Not given  **Hearing Information:**  Normal on PTA  **Reported Comorbidities:**  No depression or anxiety according to SDS/SAS. | **Sample size:**  n = 22  **Hearing:**  Normal on PTA  **Sex:**  13 F/9 M  **Age:**  44.7 ± 15.4 years  **Matched to tinnitus group for:**  Age  Sex  Hearing thresholds  Years of education | **Scanner Strength:**  3T  **Voxel Size:**  Anatomical: 1x1x1 mm^3^  Resting state: 3.75x3.75x4.0 mm^3^  **Image Acquisition:**  TR: 2000 ms  TE: 30 ms  **Instructions in Scanner:**  Subjects were instructed to lie quietly with their eyes closed without falling asleep, not think of anything in particular, and avoid any head motion during the scan. They wore ear plugs with 32 dB noise attenuation. | **Distortion Correction**  Detrending and filtering (0.01–0.08Hz)  **Software Used:**  Data Processing Assistant for Resting State fMRI (DPARSF) programs based on SPM8, and REST  **Motion Correction:**  Realignment  **Slice timing Correction:**  Yes  **Spatial Smoothing:**  Yes, Gaussian kernel FWHM = 6mm.  **Spatial Normalisation:**  MNI template | **Type of Analysis:**  Degree centrality of the whole-brain network (data-driven method for selecting ROIs) and Granger Causality Analysis for directional connectivity  **Region(s) of Interest:**  **Auditory (n=0)**  **Non-auditory (n=2)**   - L Superior Frontal Gyrus - R Superior Frontal Gyrus | L Superior Frontal Gyrus  🡪 L Orbitofrontal Cortex  🡪 L Precentral gyrus  🡪 L Posterior lobe of cerebellum  🡪 R Middle Occipital Gyrus  R Superior Frontal Gyrus  🡪 R Supplementary motor area | None found | THQ scores positively correlated with the increased effective connectivity from the left SFG to left OFC (r = 0.504, p = 0.020), and from the right SFG to right SMA (r = 0.526, p = 0.014). | - Motor network - Visual network - Frontal network - Somatosensory network |  |
| 17 | Chen et al., 2017  Tinnitus Distress is Linked to Enhanced Resting-State Functional Connectivity from the Limbic System to the Auditory Cortex | **Aim:**  To identify aberrant effective connectivity of the amygdala  and hippocampus in tinnitus patients and to determine the relationship with tinnitus characteristics.  **Inclusion Criteria:**  Chronic tinnitus (>6 months)  **Exclusion Criteria:** Pulsatile tinnitus, hyperacusis or Meniere’sdiseases or if they had a past history of severe alcoholism,smoking, head injury, stroke, Alzheimer’s disease, Parkin-son’s disease, epilepsy, major depression, or other neuro-logical or psychiatric illness, major medical illness (e.g.,cancer, anemia, and thyroid dysfunction), MRI contraindi-cations or severe visual loss.  **Study Location:**  Nanjing Medical University, China | **Sample Size:**  26  **Sex:**  17 F/9 M  **Age:**  50.2 ± 13.0 years  **Tinnitus Duration:**  44.1 ± 38.5 months    **Tinnitus Lateralisation:**  Bilateral/in the head (n=8)  Unilateral (n=18, 12 L/6 R)  **Tinnitus Severity:**  THQ: 50.0 ± 16.0  **Tinnitus Pitch:**  Not given  **Tinnitus Sound:**  Not given  **Hearing Information:**  Normal on PTA  **Reported Comorbidities:**  No depression or anxiety according to SDS/SAS. | **Sample size:**  23  **Hearing:**  Normal on PTA  **Sex:**  14 F/9 M  **Age:**  44.4 ± 15.1 years  **Matched to tinnitus group for:**  Age  Sex  Hearing thresholds  Handedness  Years of education | **Scanner Strength:**  3T  **Voxel Size:**  Anatomical: 1x1x1 mm^3^  Resting state: 3.75x3.75x4.0 mm^3^  **Image Acquisition:**  TR: 2000 ms  TE: 30 ms  **Instructions in Scanner:**  Subjects were instructed to lie quietly with their eyes closed without falling asleep, not think of anything in particular, and avoid any head motion during the scan. They wore ear plugs with 32 dB noise attenuation. | **Distortion Correction**  Detrending and filtering (0.01–0.08Hz)  **Software Used:**  Data Processing Assistant for Resting State fMRI (DPARSF) programs based on SPM8, and REST  **Motion Correction:**  Realignment  **Slice timing Correction:**  Yes  **Spatial Smoothing:**  Yes, Gaussian kernel FWHM = 4mm.  **Spatial Normalisation:**  MNI template | **Type of Analysis:**  Granger Causality Analysis  **Region(s) of Interest:**  **Auditory (n=0)**  **Non auditory (n=4)**   - L Amygdala - R Amygdala - L Hippocampus - R Hippocampus | L Amygdala  🡪 L Superior Temporal Gyrus  🡪 L Anterior Cingulate Cortex  🡪 R Angular Gyrus  🡪 L Precuneus  🡨 R Middle Frontal Gyrus  🡨 L Middle Temporal Gyrus  🡨 L Inferior Frontal Gyrus  🡨 L Postcentral Gyrus  R Amygdala  🡪 R Superior Temporal Gyrus  🡪 R Anterior Cingulate Cortex  🡪 R Middle Frontal Gyrus  🡪 R Supramarginal Gyrus  🡨 L Middle Temporal Gyrus  🡨 L Middle Frontal Gyrus  🡨 L Anterior Cingulate Cortex  🡨 R Inferior Frontal Gyrus  🡨 R Postcentral Gyrus  L Hippocampus  🡪 L Middle Temporal Gyrus  🡪 L Postcentral Gyrus  🡨 R Superior Frontal Gyrus  🡨 L Parahippocampal gyrus  🡨 L Insula  R Hippocampus  🡪 L Transverse Temporal Gyrus  🡪 R Middle Temporal Gyrus  🡪 R Postcentral Gyrus  🡨 L Middle Temporal Gyrus  🡨 L+R Middle Frontal Gyrus  🡨 L Angular Gyrus | L Amygdala   - L Cerebellum Posterior Lobe   R Amygdala  🡪 R Cerebellum Posterior Lobe  L Hippocampus  🡪 L Middle Occipital Gyrus  R Hippocampus  🡪 R Middle Occipital Gyrus | - THQ scores were positively correlated with increased connectivity from the left amygdala to left superior temporal gyrus (r = 0.570, p = 0.005), and from the right amygdala to right superior temporal gyrus (r = 0.487, p = 0.018). - Tinnitus duration was positively correlated with increased connectivity from the right hippocampus to the left transverse temporal gyrus (r = 0.452, p = 0.030). | - Limbic system - Auditory network - Default mode network - Dorsal attention network - Executive control of attention network - Somatosensory network - Motor network |  |
| 18 | Xu et al., 2019  Chronic Tinnitus Exhibits Bidirectional Functional Dysconnectivity in Frontostriatal Circuit | **Aim:**  To investigate directional connectivity of the nucleus accumbens (NAc) in chronic tinnitus and to ascertain the relationship between this connectivity and tinnitus characteristics.  **Inclusion Criteria:**  Chronic tinnitus >6 months, confirmed with authors via email  **Exclusion Criteria:**  Meniere’s disease, pulsatile tinnitus or hyperacusis, a history of severe alcoholism, smoking, head injury, stroke, Alzheimer’s disease, Parkinson’s disease, epilepsy, major depression, other neurological or psychiatric illness, major medical illnesses (e.g., cancer, anemia and thyroid dysfunction), MRI contraindications, and/or severe vision loss.  **Study Location:**  Nanjing Medical University, China | **Sample Size:**  n = 50  **Sex:**  32 F/18 M  **Age:**  50.20 ± 11.19 years  **Tinnitus Duration:**  37.42 ± 36.93 (months)  **Tinnitus Lateralisation:**  Right: 16; left: 18; bilateral or in the head: 16  **Tinnitus Severity:**  THQ: 52.19 ± 14.23  **Tinnitus Pitch:**  Not given  **Tinnitus Sound:**  Not given  **Hearing Information:**  Normal on PTA  **Reported Comorbidities:**  No depression or anxiety according to SDS/SAS. | **Sample size:**  n = 55  **Hearing:**  Normal on PTA  **Sex:**  34 F/21 M  **Age:**  46.82 ± 11.99 years  **Matched to tinnitus group for:**  Age  Sex  Hearing thresholds  Years of education | **Scanner Strength:**  3T  **Voxel Size:**  Anatomical: 1x1x1 mm^3^  Resting state: 3.75x3.75x4.0 mm^3^  **Image Acquisition:**  TR: 2000 ms  TE: 30 ms  **Instructions in Scanner:**  Participants were instructed to remain awake, keep their eyes closed, and stay motionless without thinking of anything in particular during scanning. They wore ear plugs with 32 dB noise attenuation. | **Distortion Correction**  Detrending and filtering (0.01–0.08Hz)  **Software Used:**  Data Processing Assistant for Resting State fMRI (DPARSF) programs  **Motion Correction:**  Realignment  **Slice timing Correction:**  Yes  **Spatial Smoothing:**  Yes, Gaussian kernel FWHM = 6mm  **Spatial Normalisation:**  MNI template | **Type of Analysis:**  Granger Causality Analysis  **Region(s) of Interest:**  **Auditory (n=0)**    **Non auditory (n=2)**   - L Nucleus Accumbens - R Nucleus Accumbens | L NAc  🡪 L Inferior Frontal Gyrus  🡨 R Middle Frontal Gyrus  🡨 R Middle Temporal Gyrus  R NAc  🡪 L Middle Frontal Gyrus  🡪 R Orbitofrontal Cortex  🡨 R Inferior Frontal Gyrus  🡨 R Middle Temporal Gyrus | L NAc  🡪 L Cuneus  R NAc  🡪 R Cuneus | - THQ scores were positively correlated with the increased directional connectivity from the right NAc to the left MFG (r = 0.357, p = 0.015) and from the right MFG to the left NAc (r = 0.626, p < 0.001). - Tinnitus duration was positively correlated with the increased directional connectivity from right NAc to right OFC (r = 0.599, p < 0.001). | - Frontostriatal circuit - Limbic system |  |

| **Non-seed-based resting-state fMRI studies (ALFF, ReHo, FMHC, cyclicity analysis)** | | | | | | | | | |
| --- | --- | --- | --- | --- | --- | --- | --- | --- | --- |
| **Nr** | **Study** | **Study Information** | **Tinnitus Group(s) Information:** | **Control Group(s) Information:** | **Scanning information** | **Data pre-processing** | **Data Analysis Information** | **Findings in tinnitus group compared to control group** | **Networks associated with the altered connectivity in chronic tinnitus** |
| 19 | Cai et al., 2019  Abnormal Spontaneous Neural Activity of the Central Auditory System Changes the Functional Connectivity in the Tinnitus Brain: A Resting-State Functional MRI Study | **Aim:**  To investigate abnormal functional connections between aberrant spontaneous activity in the central auditory system and the whole brain in tinnitus patients  **Inclusion Criteria:**  Tinnitus with normal hearing  **Exclusion Criteria:**  Meniere disease, conductive deafness, alternative hearing level, cognitive or mental disorders, serious systemic diseases, such as heart failure or diabetes, epilepsy, alcoholism or use of psychiatric drugs, pregnancy, acoustic neuroma, brain stem, or inferior colliculi diseases, hyperacusis, smoking, history of stroke, brain injury, Alzheimer’s disease, or Parkinson’s disease  **Study Location:**  Guangzhou, China | **Sample size:**  n = 16  **Sex:**  10 F/6 M  **Age:**  35.33 ± 10.70 years  **Tinnitus Duration:**  36.58 ± 18.03 months    **Tinnitus Lateralisation:**  Unilateral, right side (n=16)  **Tinnitus Severity:**  THI score: 55.33 ± 11.03  **Tinnitus Pitch:**  Not given  **Tinnitus Sound:**  Not given  **Hearing Information:**  Normal on PTA  **Reported Comorbidities:**  No depression and anxiety | **Sample size:**  n = 15  **Hearing:**  Normal on PTA  **Sex:**  10 F/5 M  **Age:**  35.00 ± 10.10 years  **Matched to tinnitus group for:**  Age  Sex  Years of education | **Scanner Strength:**  3T  **Voxel Size:**  Anatomical: 1x1x1 mm^3^  Resting state: 3.125x3.125x3 mm^3^  **Image Acquisition:**  TR: 2000 ms  TE: 30 ms  **Instructions in Scanner:**  All subjects were asked to remain relaxed with their eyes closed and to avoid serious thought for approximately 20 min. | **Distortion Correction**  Global signal regression & Temporal filtering (0.01–0.1 Hz) on all time-series, except for ALFF.  **Software Packages:**  DPAESF (Data Processing Assistant for Resting-state fMRI) toolbox in SPM  **Motion Correction:**  Realignment  **Slice timing Correction:**  Yes  **Spatial Smoothing:**  Yes, Gaussian kernel  FWHM = 8mm  **Spatial Normalisation:**  MNI template | **Type of Analysis:**  Smoothed mean amplitude of low-frequency fluctations (smALFF) + seed-based  **Region(s) of Interest:** (based on ALFF outcome)  **Auditory (n=1)**   - L Higher Auditory Cortex (superior temporal gyrus, BA 22) | **Increased smALFF:**  L Higher Auditory Cortex (HAC)   - Positively correlated with tinnitus duration (r = 0.778, p > 0.001), Tinnitus Handicap Inventory Score (r = 0.682, p = 0.004), and Self-Rating Depression Score (r = 0.694, p = 0.003).   **Decreased smALFF:**  R Inferior Colliculus   - Not correlated with any clinical characteristics.   Enhanced FC with HAC:   - L+R Heschl’s Gyrus - L+R Superior Temporal Gyrus - R Middle Temporal Gyrus - R Inferior Colliculus - L Cerebellum 4,5 - L Cerebellum 8 - L Hippocampus - L Amygdala - R Supramarginal Gyrus - R Insula - L+R Supplementary Motor Area   Decreased FC with L HAC:  None found  Enhanced/decreased FC with IC: None found | - Auditory network - Motor network - Dorsal attention network - Executive control network - Emotional network |
| 20 | Chen et al., 2014  Aberrant spontaneous brain activity in chronic tinnitus patients revealed by resting-state functional MRI | **Aim:**  To investigate whether aberrant spontaneous brain activity exists in chronic tinnitus patients using resting-state functional magnetic resonance imaging (fMRI) technique.  **Inclusion Criteria:**  Chronic tinnitus >6 months  **Exclusion Criteria:**  Hyperacusis, Meniere's disease, objective tinnitus, pulsatile tinnitus, severe smoking/alcoholism, stroke, brain injury, Alzheimer's disease, Parkinson's disease, epilepsy, major depression, psychiatric disorder, major medical illness e.g. cancer, severe visual impairment, MRI contraindications  **Study Location:**  Zhongda Hospital Southeast University, China | **Sample Size:**  n = 31  **Sex:**  14 F/17 M  **Age:**  41.9 ± 10.8 years  **Tinnitus Duration:**  41.0 ± 36.2 months    **Tinnitus Lateralisation:**  Bilateral/central (n=12), unilateral (n=19, 6 R/13 L)  **Tinnitus Severity:**  THQ: 100.6 ± 73.4 (range: 17.41–278.15)  **Tinnitus Pitch:**  NA  **Tinnitus Sound:**  NA  **Hearing Information:**  No hearing loss  **Reported Comorbidities:**  No included participants had accompanied symptoms such as depression and anxiety according to the Self-Rating Depression Scale (SDS) and Self Rating Anxiety Scale (SAS) (overall scores below 50, respectively) | **Sample size:**  n = 32  **Hearing:**  Normal on PTA  **Sex:**  15 F/17 M  **Age:**  46.5 ± 12.6 years  **Matched to tinnitus group for:**  Age  Sex  Hearing thresholds  Handedness  Years of education | **Scanner Strength:**  3T  **Voxel Size:**  Anatomical: 1x1x1x mm^3^  Resting state:  3.75x3.75x4.0 mm^3^  **Image Acquisition:**  TR: 2000 ms  TE: 25 ms  **Instructions in Scanner:**  Subjects were asked to rest quietly with their eyes closed but to remain awake and avoid thinking of anything particular. Subjects wore ear plugs with 32 dB attenuation | **Distortion Correction**  Detrending and filtering (0.01 - 0.08 Hz)  **Software Packages:**  SPM8, REST  **Motion Correction:**  Realignment  **Slice timing Correction:**  Yes  **Spatial Smoothing:**  Yes, Gaussian kernel  FWHM = 4 mm  **Spatial Normalisation:**  MNI template | **Type of Analysis:**  Amplitude of low-frequency fluctuations (ALFF) | **Increased ALFF:**   - R Middle Temporal Gyrus - R Superior Frontal Gyrus - R Angular Gyrus   **Decreased ALFF:**   - L Cuneus - R Middle Occipital Gyrus - L+R Thalamus   The ALFF value in right SFG was positively correlated with tinnitus duration and tinnitus handicap questionnaire (THQ) score, respectively (r = 0.464, p = 0.010; r = 0.557, p = 0.007).  The ALFF value in right MTG was also positively correlated with the THQ score (r = 0.504, p = 0.004).  However, no significant correlations survived after Bonferroni correction. | - Auditory network - Default mode network - Visual network |
| 21 | Chen et al, 2015  Frequency-specific alternations in the amplitude of low-frequency fluctuations in chronic tinnitus | **Aim:**  To investigate the role of frequency-specific components of low-frequency oscillations in tinnitus using amplitude of low-frequency fluctuation (ALFF) and fractional ALFF (fALFF) in two different frequency bands (slow-4: 0.027-0.073 Hz and slow-5: 0.01-0.027 Hz).  **Inclusion Criteria:**  Chronic tinnitus (>6 months)  **Exclusion Criteria:**  Pulsatile tinnitus, hyperacusis or Meniere’s diseases, a past history of severe smoking, alcoholism, brain injury, stroke, Alzheimer’s disease, Parkinson’s disease, epilepsy, major depression, or other neurological or psychiatric disorders that could affect cognitive function, major medical illness (e.g., anaemia, thyroid dysfunction and cancer), MRI contraindications, or severe visual loss.  **Study Location:**  Zhongda Hospital of Southeast University, China | **Sample Size:**  n = 39  **Sex:**  15 F/24 M  **Age:**  41.5 ± 14.6 years  **Tinnitus Duration:**  36.9 ± 36.4 months    **Tinnitus Lateralisation:**  Bilateral (n=10)  Unilateral (n=29, 16 L/13 R)  **Tinnitus Severity:**  THQ = 43.5 ± 21.3  **Tinnitus Pitch:**  Not given  **Tinnitus Sound:**  Not given  **Hearing Information:**  Normal on PTA  **Reported Comorbidities:**  None reported | **Sample size:**  n = 41  **Hearing:**  Normal on PTA  **Sex:**  20 F/21 M  **Age:**  46.0 ± 12.2 years  **Matched to tinnitus group for;**  Age  Sex  Hearing thresholds  Years of education | **Scanner Strength:**  3T  **Voxel Size:**  Anatomical: 0.977x0.977x1 mm^3^  Resting state: 3,75x3,75x4 mm^3^  **Image Acquisition:**  TR: 2000 ms  TE: 25 ms  **Instructions in Scanner:**  Subjects were instructed to lie quietly with their eyes closed without falling asleep, not think of anything in particular, and avoid any head motion during the scan | **Distortion Correction**  Detrending and filtering (0.01 - 0.08 Hz)  **Software Used:**  Data Processing Assistant for Resting State fMRI (DPARSF) programs based on SPM8, and REST  **Motion Correction:**  Realignment  **Slice timing Correction:**  Yes  **Spatial Smoothing:**  Yes, Gaussian kernel  FWHM = 6 mm  **Spatial Normalisation:**  MNI template | **Type of Analysis:**  Amplitude of low-frequency fluctuation (ALFF) and fractional ALFF (fALFF) | **Increased ALFF:**   - R Superior Frontal Gyrus - R Middle Temporal Gyrus - R Angular Gyrus - L Inferior Frontal Gyrus - R Supramarginal Gyrus   **Decreased ALFF:**   - L+R Middle Occipital Gyrus   **Increased fALFF:**   - L Superior Frontal Gyrus - R Supramarginal Gyrus   **Decreased fALFF:**   - L+R Middle Occipital Gyrus   Slow-4 ALFF values in R SFG and fALFF values in L SFG were positively correlated with THQ scores (respectively, r = 0.446, p = 0.007; r = 0.466, p = 0.005).  Slow-5 ALFF values in R SFG and fALFF values in L SFG were positively correlated with tinnitus duration (respectively, r = 0.544, p = 0.001; r = 0.526, p = 0.001). | - Auditory network - Default mode network - Visual network |
| 22 | Han et al, 2018  Disrupted local neural activity and functional connectivity in subjective tinnitus patients: evidence from resting-state fMRI study | **Aim:**  To investigate the abnormal alterations of both the intra-regional brain activity and inter-regional  functional connectivity (FC) in patients with subjective tinnitus using resting-state functional MRI (rs-fMRI) methods.  **Inclusion Criteria:**  Chronic subjective tinnitus (> 6 months)  **Exclusion Criteria:**  Hyperacusis or Meniere’s disease, neurological or psychiatric disorders, and any contraindication for MRI scans.  **Study Location:**  Southwest Hospital, Third Military Medical University (Army Medical University), Chongqing, China | **Sample Size:**  n = 25  **Sex:**  15 F/10 M  **Age:**  44.64 ± 8.91 years  **Tinnitus Duration:**  Median = 14.00 (8.50–54.00) months    **Tinnitus Lateralisation:**  All unilateral (8 L/17 R)  **Tinnitus Severity:**  THI: 47.16 ± 24.95  **Tinnitus Pitch:**  Not given  **Tinnitus Sound:**  Not given  **Hearing Information:**  Normal on PTA  **Reported Comorbidities:**  Not given | **Sample size:**  n = 25  **Hearing:**  Normal on PTA  **Sex:**  15 F/10 M  **Age:**  43.96 ± 8.92 years  **Matched to tinnitus group for:**  Age  Sex  Hearing thresholds  Years of education  Hyperacusis questionnaire score | **Scanner Strength:**  3T  **Voxel Size:**  Anatomical: 1x1x1 mm^3^  Resting state: 3×3×3 mm^3^  **Image Acquisition:**  TR: 2000 ms  TE: 30 ms  **Instructions in Scanner:**  Subjects were asked to keep their eyes closed but remain awake, rest quietly, and avoid thinking of anything in particular | **Distortion Correction**  Nuisance covariates regression  Band-pass filtering (0.01 - 0.08 Hz)  **Software Used:**  Data Processing & Analysis for Brain Imaging (DPABI) based on Matrix Laboratory and SPM8  **Motion Correction:**  Realignment  **Slice timing Correction:**  Yes  **Spatial Smoothing:**  Yes, Gaussian kernel  FWHM = 4mm  **Spatial Normalisation:**  MNI template | **Type of Analysis:**  Regional homogeneity (ReHo), fALFF, seed-based FC  **Seed-based ROIs:** (selected based on ReHo & fALFF results)   - R Middle Temporal Gyrus - R Cuneus - R Middle Frontal Gyrus - L Cerebellar Anterior Lobe | **Increased ReHo**   - R Middle Temporal Gyrus - R Cuneus   **Decreased ReHo:**   - R Middle Frontal Gyrus - L Cerebellar Anterior Lobe   **Increased fALFF:** - R Middle Temporal Gyrus  **Decreased fALFF:**  None found  **Increased FC:**  None found  **Decreased FC:**  R Middle Temporal Gyrus   - R Middle Frontal Gyrus* - R Lingual Gyrus - R Cerebellar Posterior Lobe   R Cuneus   - R MTG   * Positively correlated with Tinnitus Handicap Inventory score (r = 0.675, p = 0.001). | - Auditory network - Default mode network - Visual network |
| 23 | Chen et al, 2015  Altered intra- and interregional synchronization in resting-state cerebral networks associated with chronic tinnitus | **Aim:**  To identify aberrant neural networks involved in chronic tinnitus, by comparing the resting-state functional magnetic resonance  imaging (fMRI) patterns of tinnitus patients and healthy controls.  **Inclusion Criteria:**  Chronic tinnitus (>6 months), confirmed with authors via email  **Exclusion Criteria:**  Hyperacusis, pulsatile tinnitus, or Meniere’s diseases or a past history of severe smoking, stroke, alcoholism, brain injury, Parkinson’s disease, Alzheimer’s disease, epilepsy, major depression, neurological or psychiatric disorders that could affect cognitive function, major medical illness (e.g., anemia, thyroid dysfunction, and cancer), MRI contraindications, or severe visual loss.  **Study Location:**  Zhongda Hospital Southeast University, China | **Sample Size:**  n = 29  **Sex:**  13 F/16 M  **Age:**  40.9 ± 10.5 years  **Tinnitus Duration:**  39.5 ± 33.7 months  **Tinnitus Lateralisation:**  Bilateral/in the head (n=11)  Unilateral (n=18, 12 L/6 R)  **Tinnitus Severity:**  THQ: 103.5 ± 74.4  **Tinnitus Pitch:**  Not given  **Tinnitus Sound:**  Not given  **Hearing Information:**  Normal on PTA  **Reported Comorbidities:**  No depression or anxiety according to SDS/SAS. | **Sample size:**  n = 30  **Hearing:**  Normal on PTA  **Sex:**  15 F/15 M  **Age:**  46.2 ± 11.9 years  **Matched to tinnitus group for;**  Age  Sex  Hearing thresholds  Years of education | **Scanner Strength:**  3T  **Voxel Size:**  Anatomical: 0,98x0,98x1 mm^3^  Resting state: 0,98x0,98x1 mm^3^  **Image Acquisition:**  TR: 2000 ms  TE: 25 ms  **Instructions in Scanner:**  Subjects were instructed to lie quietly with their eyes closed without falling asleep, not think of anything in particular, and avoid any head motion during the scan. They wore ear plugs with 32 dB noise attenuation. | **Distortion Correction**  Detrending and filtering (0.01–0.08Hz)  **Software Used:**  Data Processing Assistant for Resting State fMRI (DPARSF) programs based on SPM8, and REST  **Motion Correction:**  Realignment  **Slice timing Correction:**  Yes  **Spatial Smoothing:**  Yes, Gaussian kernel  FWHM = 4mm  **Spatial Normalisation:**  MNI template | **Type of Analysis:**  Regional homogeneity (ReHo) + seed-based FC  **Seed-based ROIs: (s**elected based on ReHo results)  **Auditory (n=0)**  **Non auditory (n=4)**   - L Anterior Insular Cortex - R Anterior Insular Cortex - L Inferior Frontal Gyrus - R Supramarginal Gyrus | **Increased ReHo:**   - L Anterior Insular Cortex - R Anterior Insular Cortex - L Inferior Frontal Gyrus - R Supramarginal Gyrus   **Decreased ReHo:** - L Cuneus  **Increased FC:**  L Anterior Insular Cortex   - L Middle Frontal Gyrus* - R Inferior Temporal Gyrus - R Precuneus   R Anterior Insular Cortex   - R Middle Frontal Gyrus** - R Superior Temporal Gyrus - L Precuneus - L Posterior Cingulate Cortex   L Inferior Frontal Gyrus   - R Middle Frontal Gyrus - R Inferior Temporal Gyrus - R Anterior Cingulated Cortex   R Supramarginal Gyrus   - L Inferior Frontal Gyrus - R Orbitofrontal Cortex   **Decreased FC:**  None found  * Positively correlated with THQ score (r = 0.459, p = 0.012).  ** Positively correlated with THQ score (r = 0.479, p = 0.009). | - Executive control of attention network - Default mode network - Visual network |
| 24 | Gentil et al, 2019  Alterations in Regional Homogeneity in Patients With Unilateral Chronic Tinnitus | **Aim:**  To identify differences in cerebral ReHo in patients with unilateral tinnitus compared with non-tinnitus control subjects in a resting state. Our second objective is to highlight lateralized differences related to tinnitus lateralization.  **Inclusion Criteria:**  Chronic tinnitus, >1 year  **Exclusion Criteria:**  Neurological disorders, Meniere’s disease, temporo-mandibular joint disorders, and other neurological issues or chronic physical diseases  **Study Location:**  Montpellier University Hospital, France. | **Sample Size:**  n = 19  **Sex:**  5 F/14 M  **Age:**  63 ± 10 years  **Tinnitus Duration:**  12 ± 13 years  **Tinnitus Lateralisation:**  unilateral 10 right, 9 left  **Tinnitus Severity:**  THI = 36 ± 13  **Tinnitus Pitch:**  4 kHz n=9  6 kHz, n=10  **Tinnitus Sound:**  Not given  **Hearing Information:**  Average PTA hearing thresholds were in the normal range for the control group whereas the thresholds were moderately elevated in the tinnitus group for 4 & 6 kHz (above 6 kHz not tested).  **Reported Comorbidities:**  Not given;  Presence/absence of hyperacusis not mentioned. | **Sample size:**  n = 16  **Hearing:**  Average hearing thresholds in PTA are in normal range  **Sex:**  9 F/7 M  **Age:**  59 ± 11 (range 39–78) years  **Matched to tinnitus group for:**  Age  Handedness  Years of education  Not matched for sex  Not matched for hearing thresholds >4 kHz | **Scanner Strength:**  3T  **Voxel Size:**  Anatomical: 1x1x1 mm^3^  Functional: 3.8 × 3.8 × 3 mm^3^  Resting state: 2.39 x 2.39 x 3 mm^3^  **Image Acquisition:**  TR: 2400 ms  TE: 30 ms  **Instructions in Scanner:**  Keep eyes closed, think about nothing in particular, and do not fall asleep | **Distortion Correction**  Field map correction  Detrending and filtering (0.01–0.08Hz)  **Software Used:**  SPM 12, REST  **Motion Correction:**  Realignment  **Slice timing Correction:**  Yes  **Spatial Smoothing:**  Yes, Gaussian kernel  FWHM = 6mm  **Spatial Normalisation:**  MNI template | **Type of Analysis:**  Regional homogeneity (ReHo) + correlation analysis | **Increased ReHo:**  None found  **Decreased ReHo:**   - Cluster between STG/MTG (auditory cortex), contralateral to tinnitus ear   **Correlation between ReHo values in brain regions and clinical characteristics (p<0.005):**   - STG/MTG (contralateral) & Visual Analogue Scale for tinnitus loudness (r = -0.6156); THI score (r = -0.6336) - Lingual Gyrus (contralateral) & Visual Analogue Scale for tinnitus loudness (r = -0.5806) - Precentral Gyrus (ipsilateral) & THI score (r = -0.5537) - Middle Temporal Gyrus (ipsilateral) & HHTP (hearing threshold at tinnitus pitch) (r = 0.83704) - Supramarginal/angular Gyrus (ipsilateral) & HTTP (r = 0.7739); tinnitus duration (r = 0.7344) - Lingual Gyrus (ipsilateral) & tinnitus duration (r = 0.7974) - Superior Frontal Lobe (ipsilateral) & tinnitus duration (r = 0.7898) | - Auditory network |
| 25 | Chen et al, 2015  Altered interhemispheric functional coordination in chronic tinnitus patients | **Aim:**  To examine the resting-state  interhemispheric functional connectivity and its relationships with clinical characteristics in chronic tinnitus patients using a novel method, voxel-mirrored homotopic connectivity (VMHC).  **Inclusion Criteria:**  Chronic tinnitus (>6 months)  **Exclusion Criteria:**  Hyperacusis, pulsatile tinnitus, or Meniere’s diseases or a past history of severe smoking, stroke, alcoholism, brain injury, Parkinson’s disease, Alzheimer’s disease, epilepsy, major depression, neurological or psychiatric disorders that could affect cognitive function, major medical illness (e.g., anemia, thyroid dysfunction, and cancer), MRI contraindications, or severe visual loss.  **Study Location:**  Zhongda Hospital Southeast University, China | **Sample Size:**  n = 28  **Sex:**  12 F/16 M  **Age:**  40.5 ± 13.2 years  **Tinnitus Duration:**  34.3 ± 34.2 months    **Tinnitus Lateralisation:**  Bilateral or in the head (n=7)  Unilateral (n=21, 9 R/12 L)  **Tinnitus Severity:**  THQ: 41.3 ± 18.2  **Tinnitus Pitch:**  Not given  **Tinnitus Sound:**  Not given  **Hearing Information:**  **No hearing loss**  Normal on PTA  **Reported Comorbidities:**  No depression or anxiety according to SDS/SAS. | **Sample size:**  n = 30  **Hearing:**  Normal on PTA  **Sex:**  15 F/15 M  **Age:**  46.2 ± 11.9 years  **Matched to tinnitus group for;**  Age  Sex  Hearing thresholds  Years of education | **Scanner Strength:**  3T  **Voxel Size:**  0,98x0,98x1 mm^3^  3,75x3,75x4 mm^3^  **Image Acquisition:**  TR: 2000 ms  TE: 25 ms  **Scanner instructions:**  Subjects were instructed to lie quietly with their eyes closed without falling asleep, not think of anything in particular, and avoid any head motion during the scan. They wore ear plugs with 32 dB noise attenuation. | **Distortion Correction**  Detrending and filtering (0.01–0.08Hz)  **Software Used:**  Data Processing Assistant for Resting State fMRI (DPARSF) programs based on SPM8, and REST  **Motion Correction:**  Realignment  **Slice timing Correction:**  Yes  **Spatial Smoothing:**  Yes, Gaussian kernel  FWHM = 6mm.  **Spatial Normalisation:**  MNI template | **Type of Analysis:**  Voxel-mirrored homotopic connectivity (VMHC) + correlation analysis  **Region(s) of Interest:**  **Auditory (n=0)**  **Non auditory (n=1)**   - Interhemispheric connections and the corpus Collosum | **Increased VMHC:**   - Middle Temporal Gyrus - Middle Frontal Gyrus - Superior Occipital Gyrus   **Decreased VMHC:**  None found  **Correlation between VMHC values in brain regions and clinical characteristics (p<0.05):**   - L+R Uncus & tinnitus duration (r = 0.62026) - L+R Transverse Temporal Gyrus (BA 42, secondary auditory cortex) & THQ score (r = 0.63775) - L+R Superior Temporal Pole & THQ score (r = 0.71195) - L+R Precentral Gyrus & THQ score (r = 0.64225) - L+R Calcarine Cortex & THQ score (r = 0.65234) | - Auditory network - Visual network - Motor network - Default mode network - Limbic system |
| 26 | Zimmerman et al, 2019  Dissociating tinnitus patients from healthy controls using resting-state cyclicity analysis and clustering | **Aim:**  To explore leader-follower patterns in the temporal ordering of resting-state fMRI data using a novel analysis method, cyclicity analysis, and to explore different machine learning classification methods to differentiate between tinnitus and control populations.  **Inclusion Criteria:**  Chronic tinnitus, >6 months, confirmed via email  **Exclusion Criteria:**  Not given  **Study Location:**  Illinois, University of Illinois Urbana-Champaign, USA | **Sample Size:**  n = 32  **Sex:**  14 F/18 M  **Age:**  51.15 ± 10.73 years  **Tinnitus Duration:**  Not given  **Tinnitus Lateralisation:**  Not given  **Tinnitus Severity:**  TFI: 23.44 ± 17.78  **Tinnitus Pitch:**  Not given  **Tinnitus Sound:**  Not given  **Hearing Information:**  Mild to moderate bilateral high-frequency hearing loss  **Reported Comorbidities:**  BDI: no to minimal depression | **Sample size:**  n = 15  **Hearing:**  PTA average thresholds up to 8 kHz in normal range, thresholds slightly elevated above 8 kHz.  **Sex:**  10 F/5 M  **Age:**  47.27 (SD 11.71) years  **Matched to tinnitus group for;**  Age  BDI and BAI scores  Not matched for sex  Not matched for hearing thresholds >3kHz | **Scanner Strength:**  3T  **Voxel Size:**  **Anatomical:** 0.9x0.9x0.9 mm^3^  **Resting state:** 2.5x2.5x3.0 mm^3^  **Image Acquisition:**  TR: 2000 ms  TE: 25 ms  **Instructions in Scanner:**  Lie still with eyes open fixated on a cross, do not think about anything in particular | **Distortion Correction**  Not given  **Software Used:**  SPM12  **Motion Correction:**  six-parameter rigid body transformation for head motion correction  **Slice timing Correction:**  Yes  **Spatial Smoothing:**  Yes, Gaussian kernel  FWHM = 8mm.  **Spatial Normalisation:**  MNI template | **Type of Analysis:**  Cyclicity Analysis  **Region(s) of Interest:**  **Auditory (n=2)**   - R primary auditory cortex - L primary auditory cortex   **Non auditory (n=31)**   - L amygdala - L anterior insula - L cuneus - L frontal eye field - L inferior frontal lobe - L inferior parietal lobe - L mid frontal gyrus - L parahippocampus - L posterior intraparietal sulcus - L primary visual cortex - L superior occipital lobe - L superior temporal junction - L superior temporal sulcus - L ventral intraparietal sulcus - Medial prefrontal cortex - Posterior cingulate cortex - Precuneus - R amygdala - R anterior insula - R cuneus - R frontal eye field - R inferior frontal lobe - R inferior parietal lobe - R mid frontal gyrus - R parahippocampus - R posterior intraparietal sulcus - R primary visual cortex - R superior occipital lobe - R superior temporal junction - R superior temporal sulcus   R ventral intraparietal sulcus | Cyclicity analysis was able to differentiate between TIN and Control groups with 58-67% accuracy.  In the controls, there were consistent temporal patterns across frontal, parietal, and limbic regions and amygdalar activity, whereas in tinnitus subjects, this pattern was much more variable.  The 20 ROI pairs that helped most to distinguish between tinnitus and control participants were:   1. Precuneus 2. L Posterior Intraparietal Sulcus 3. R Postrior Intraparietal Sulcus 4. R Ventral Intraparietal Sulcus 5. L Inferior Parietal Lobule 6. L Superior Occipital Lobe 7. R Superior Occipital Lobe 8. R Primary Visual Cortex 9. L Frontal Eye Field 10. R Frontal Eye Field 11. L Middle Frontal Gyrus 12. R Middle Frontal Gyrus 13. Medial Prefrontal Cortex 14. L Primary Auditory Cortex 15. L Superior Temporal Junction 16. R Superior Temporal Junction 17. Posterior Cingulate Cortex 18. L Parahippocampus 19. R Parahippocampus 20. L Amygdala | - Auditory network - Default mode network - Dorsal attention network - Visual network - Attention control network - Ventral attention network - Limbic system |

| **Data-driven resting-state fMRI studies: Independent Component Analysis** | | | | | | | | | | |
| --- | --- | --- | --- | --- | --- | --- | --- | --- | --- | --- |
| **Nr** | **Study** | **Study Information** | **Tinnitus Group(s) Information:** | **Control Group(s) Information:** | **Scanning Information** | **Data Pre-processing** | **Data Analysis Information** | **Regions showing increased connectivity in tinnitus compared to controls** | **Regions showing decreased connectivity in tinnitus compared to controls** | **Networks associated with the altered connectivity in chronic tinnitus** |
| 27 | Davies et al, 2014  Auditory network connectivity in tinnitus patients: A resting-state fMRI study | **Aim:**  To investigate auditory network connectivity, adopting and extending previously used analyses methods to provide an independent evaluation of replicability.  **Inclusion Criteria:**  Tinnitus duration >2 years  **Exclusion Criteria:**  Unilateral/asymmetrical hearing loss; hyperacusis  **Study Location:**  Queen’ s Medical Centre, Nottingham, UK | **Sample Size:**  n = 12  **Sex:**  5 F/7 M  **Age:**  65.8 (range: 49-73) years, SD not given  **Tinnitus Duration:**  15.5 ± 20.4 years    **Tinnitus Lateralisation:**  Bilateral (n=7)  Central (n=3)  Unilateral (n=2, 1 L/1 R)  **Tinnitus Severity:**  THQ: 43.7 ± 18.32  **Tinnitus Pitch:**  Not given  **Tinnitus Sound:**  Not given  **Hearing Information:**  Mild to moderately severe sloping hearing loss, typical of presbyacusis.  **Reported Comorbidities:**  No depression or anxiety according to BDI and BAI. | **Sample size:**  n = 11  **Hearing:**  **Sex:**  3 F/8 M  **Age:**  68.5 years (range 58-75). SD not given  **Matched to tinnitus group for:**  Age  Sex  Hearing thresholds  BAI & BDI scores | **Scanner Strength:**  3T  **Voxel Size:**  Anatomical: 1x1x1 mm^3^  Resting state: 3.75x3.75x4.0 mm^3^  **Image Acquisition:**  TR: 2700 ms  TE: 20, 45 ms (two echo pulses)  **Instructions in Scanner:**  Keep still and alert with your eyes closed. Participant wore ear plugs as well as circum-aural active noise cancelling headphones. | **Distortion Correction:**  Not given  **Software Used:**  SPM8  **Motion Correction:**  Realignment  **Slice timing Correction:**  No  **Spatial Smoothing:**  Yes, Gaussian kernel  FWHM = 4mm  **Spatial Normalisation:**  MNI template | **Type of Analysis:**  Independent Component Analysis (ICA) + seed-based FC  **Region(s) of Interest:** (selected based on ICA)    **Auditory (n=4)**  Concatenated to form one “Auditory Component”:   - L Primary Auditory Cortex - R Primary Auditory Cortex - L Secondary Auditory Cortex - R Secondary Auditory Cortex   **Non-auditory (n=0)** | None found: result below did not survive after correcting for multiple comparisons.  Auditory component   - R Supramarginal Gyrus - L Posterior Middle Temporal Gyrus | None found | None found |
| 28 | Maudoux et al, 2012  Auditory Resting-State Network Connectivity in Tinnitus: A Functional MRI Study | **Aim:**  The aim of this study was to test if functional MRI resting-state connectivity patterns in the auditory network differ between tinnitus patients and normal controls.  **Inclusion Criteria:**  Chronic tinnitus present either constantly or intermittently for at least 1 year.  **Exclusion Criteria:**  Major neurological, neurosurgical or psychiatric history, hyperacusis, phonophobia.  **Study Location:**  University of Liege, Belgium | **Sample Size:**  n = 13  **Sex:**  6 F/7 M  **Age:**  52 ± 11 years  **Tinnitus Duration:**  8 ± 9 years  **Tinnitus Lateralisation:**  Bilateral (n=3)  Unilateral (n=10, 6 L/4 R)  **Tinnitus Severity:**  Slight to catastrophic  THI/TQ: 43.5/31.9.  **Tinnitus Pitch:**  M = 4846 Hz (SD = 2276), ranging from 1500 Hz - 8000 Hz  **Tinnitus Sound:**  Not given  **Hearing Information:**  4 patients had normal hearing; the rest ranged from mild to severe hearing loss (n=9).  **Reported Comorbidities:**  Not given. Anxiety/depression not tested. | **Sample size:**  n = 27  Control1: 12 (for auditory component selection)  Control2: 15 (for comparing to tinnitus group)  **Hearing:**  Control1: not tested  Control2: not given  **Sex:**  Control1: 4 F/8 M  Control2: 6 F/9 M  **Age:**  Control1: 21 ± 3  Control2: 51 ± 13  **Matched to tinnitus group for;**  Control1 not matched  Control2 matched for age and sex;  Not matched for hearing thresholds. | **Scanner Strength:**  3T  **Voxel Size:**  Tinnitus: 3.0x3.0x3.75 mm^3^  Control: 3.4x3.4x3.0 mm^3^  **Image Acquisition:**  Tinnitus  TR: 2000 ms  TE: 30 ms  Control  TR: 2460ms  TE: 40ms  **Instructions in Scanner:**  Not given | **Distortion Correction**  Filtering out low frequencies of up to 0.005 Hz and linear trend removal  **Software Used:**  Brain Voyager  **Motion Correction:**  Realignment  **Slice timing Correction:**  Yes  **Spatial Smoothing:**  Yes, Gaussian kernel  FWHM = 8mm  **Spatial Normalisation:**  Talairach and Tournoux (1988) standard anatomical space | **Type of Analysis:**  Independent Component Analysis + seed-based FC  **Region(s) of Interest:**  (selected based on ICA)  **Auditory (n=14)**  Combined into one “Auditory Component”   - 3x R Transverse Temporal Gyrus - 3x L Transverse Temporal Gyrus - 3x R Superior Temporal Gyrus - 3x L Superior Temporal Gyrus - R Insula - L Insula | Auditory Component   - L+R Parahippocampal Gyrus - L+R Brainstem/Cerebellum - L Precentral Gyrus - L Superior Temporal Gyrus - L Inferior Frontal Gyrus - R Basal Ganglia/Nucleus Accumbens - R Prefrontal cortex - L Postcentral Cyrus - R Orbitofrontal Cortex - R Inferior Parietal Lobe | Auditory Component   - L Superior Frontal Gyrus - L Fusiform Gyrus - R Superior Temporal Gyrus - R Occipital Cortex - L Occipital Cortex - L Prefrontal Cortex | - Auditory network - Attentional network - Memory network - Emotional network - Visual network |
| 29 | Schmidt et al, 2013  Default mode, dorsal attention and auditory resting state networks exhibit differential functional connectivity in tinnitus and hearing loss | **Aim:**  To investigate auditory, dorsal attention, and default mode networks in adults with tinnitus and hearing loss in a resting state functional connectivity study.  **Inclusion Criteria:**  Not given  **Exclusion Criteria:**  Not given  **Study Location:**  Illinois, USA | **Sample Size:**  n = 12  **Sex:**  3 F/9 M  **Age:**  55.00 ± 6.97  **Tinnitus Duration:**  Not given  **Tinnitus Lateralisation:**  Not given  **Tinnitus Severity:**  THI = 8.33 ± 6.76  **Tinnitus Pitch:**  Not given  **Tinnitus Sound:**  Not given  **Hearing Information:**  Hearing loss was minimal from 250-2000 Hz and moderate to moderate-severe (threshold ≥ 35 dB) for 3-8 kHz  **Reported Comorbidities:**  No hyperacusis | **Sample size:**  NH controls: n=15  HL controls: n=13  **Hearing:**  NH: normal on PTA  HL: minimal from 250-2000 Hz and moderate to moderate-severe (threshold ≥ 35 dB) for 3-8 kHz  **Sex:**  NH:  6 F/9M  HL:  8 F/5M  **Age:**  NH: 52.93 ± 8.64  HL: 57.62 ± 9.39  **Matched to tinnitus group for:**  NH:  Age  Sex  HLs: Age  Sex  Hearing thresholds | **Scanner Strength:**  3T  **Voxel Size:**  Anatomical: 1.0×1.0×1.2 mm^3^  Resting state: 3.4×3.4×4.0 mm^3^  **Image Acquisition:**  TR: 2000 ms  TE: 30 ms  **Instructions in Scanner:**  Subjects were instructed to lay still and look at a fixation cross. | **Distortion Correction**  Band-pass filtering (0.008 – 0.08 Hz)  **Software Used:**  SPM8, CONN, GIFT software  **Motion Correction:**  Realignment  **Slice timing Correction:**  Yes  **Spatial Smoothing:**  Yes, Gaussian kernel  FWHM = 10mm  **Spatial Normalisation:**  MNI template | **Type of Analysis:**  Independent Component Analysis + seed-based FC  **Region(s) of Interest:**    **Auditory component (n=2)**   - L Primary Auditory Cortex - R Primary Auditory Cortex   **Dorsal Attention Network component #1 (n=2)**   - L Posterior Intraparietal Sulcus - R Posterior Intraparietal Sulcus   **Dorsal Attention Network component #2 (n=2)**   - L Frontal Eye Field - R Frontal Eye Field   **Default Mode Network component (n=2)**   - Medial Prefrontal Cortex - Posterior Cingulate Cortex | Auditory component   - L Lingual Gyrus (TIN>NH) - L Parahippocampus (TIN>NH)   DAN #2 component   - R Parahippocampus (TIN>HL)   DMN component   - R Fusiform Gyrus (TIN>HL) - R Lingual Gyrus (TIN>HL) | DAN #1 component   - R Supramarginal Gyrus (HL>TIN)   DMN component   - L Precuneus (HL>TIN) - L Precentral Gyrus (HL>TIN) - L Cerebellum (HL>TIN) - L Cerebellar Vermis (HL>TIN) - R Precuneus (NH>TIN) | - Auditory network - Dorsal attention network - Default mode network - Motor network - Limbic system |
